# An observational retrospective study of online owner perceptions of diet-related health signs in dogs using social media listening

**DOI:** 10.1093/jvimsj/aalag245

**Published:** 2026-10-26

**Authors:** Jasmeen P Kaur, Teresa Hollands, Ciara Clarke, Georgina Tarrant, Taran Rai, Travis Street, Kevin Wells, Susan K Armstrong

**Affiliations:** School of Veterinary Medicine, University of Surrey, Guildford GU2 7AL, United Kingdom; School of Veterinary Medicine, University of Surrey, Guildford GU2 7AL, United Kingdom; Dogmates UK Ltd, London W12 7RA, United Kingdom; Surrey DataHub, vHive, School of Veterinary Medicine, University of Surrey, Guildford GU2 7AL, United Kingdom; Surrey DataHub, vHive, School of Veterinary Medicine, University of Surrey, Guildford GU2 7AL, United Kingdom; Surrey DataHub, vHive, School of Veterinary Medicine, University of Surrey, Guildford GU2 7AL, United Kingdom; Surrey DataHub, vHive, School of Veterinary Medicine, University of Surrey, Guildford GU2 7AL, United Kingdom; School of Veterinary Medicine, University of Surrey, Guildford GU2 7AL, United Kingdom

**Keywords:** food intolerance, fresh diets, kibble diet, large language models, nutrition, topic modeling

## Abstract

**Background:**

Dog owners increasingly link health to diet type, and the pet food market has diversified beyond kibble/canned diets (KC) to raw and fresh diets (FD; human-grade, gently/mildly cooked, and home-cooked). However, it remains unclear which owner-reported health-related signs (ORHS) are associated with dietary changes, including KC-FD transitions. Social media listening (SML) enables large-scale capture of naturally-occurring online owner discussions.

**Hypothesis/Objectives:**

Investigate owner perceptions of diet-related ORHS in dogs within online discourse using SML.

**Animals:**

None.

**Methods:**

Observational, retrospective study of 21 896 KC and 2336 FD online posts (>96% from Reddit) collected using Pulsar Platform™ (January 1, 2025 to July 3, 2025). After preprocessing and large language model (LLM)-based zero-shot filtering, 8040 KC and 924 FD posts were analyzed using LLM-assisted topic modeling and dictionary-based ORHS quantification.

**Results:**

Topic modeling identified recurrent themes across KC (number of topics [k] = 7, coherence score [Cv] = 0.58) and FD (k = 6, Cv = 0.44) posts. Owners discussed diet as both the perceived cause and management strategy for gastrointestinal (GI), dermatologic, allergy/intolerance, appetite, and body condition ORHS, alongside decision uncertainties. Gastrointestinal (2286/8040, 28.4%; 282/924, 30.5%) and allergy/intolerance (1932/8040, 24.0%; 194/924, 21.0%) ORHS predominated within KC and FD posts, respectively; allergy/intolerance co-occurred with skin/coat (478/918, 52.1%; 57/114, 50.0%), and GI with allergy/intolerance (476/1,932, 24.6%; 68/194, 35.1%).

**Conclusions and clinical importance:**

Our study demonstrates the feasibility of SML-LLM-assisted analysis for capturing diet-related ORHS in online dog owner discourse. The study captured owner perceptions rather than clinically verified outcomes, highlighting the need for future studies to align ORHS with clinically validated endpoints to support veterinary nutrition discussions and informed owner decision-making.

## Introduction

Nutrition plays a critical role in the prevention and management of diseases, modulating gut microbial diversity and metabolic and inflammatory processes associated with chronic conditions such as obesity and skin disease.^1–5^ Dietary choices are shaped by owner perceptions and beliefs, with commercial diets, including over-the-counter formulations, typically selected for convenience, cost, and palatability, whereas alternative diets (eg, raw or low-carbohydrate) are often adopted to address perceived health concerns, including coat condition, pruritus, and gastrointestinal (GI) health. ^6–13^ Although most owners feed kibble/canned diets (KC; 79%), inclusion of raw and homemade diets has increased over the last decade (16% to 66% and 31% to 64%, respectively), whereas exclusive feeding remains uncommon (9% raw, 3.6% homemade), reflecting diversification of feeding practices.^14^

Raw and fresh diets (FD) have been shown to alter the gut microbiome, and although owners frequently report perceived health benefits with raw feeding, robust scientific evidence, and clear biological mechanisms linking these diets to reported outcomes remain uncertain.^10–12^^,^^15^^,^^16^ Similarly, FD are marketed with claims relating to human-grade ingredients, nutrient integrity, and absence of preservatives, but owner perceptions and evidence supporting such claims are not well understood.^17^

Veterinarians recognize nutrition as central to preventative medicine, reflected in its inclusion in routine history taking, but many report limited confidence in providing dietary advice, partly due to a lack of comparative, evidence-based data across diet types and their impact on dog health.^18–20^ Most studies exploring owner perceptions have relied primarily on surveys to capture owner-reported experiences.^10^^,^^12^^,^^21^^,^^22^ Although valuable, survey-based approaches are limited by recall bias, self-selection bias, and are reliant on pre-defined response frameworks, which may overlook unsolicited, real-world experiences.

Social media listening (SML) offers a complementary approach, analyzing large volumes of naturally-occurring online discussions to capture owner perspectives in real time, without researcher prompting. This method decreases recall and recruitment bias, captures diverse perspectives, and enables early identification of emerging themes. Social media listening has been successfully applied in contexts such as owner challenges with oral medication, role of women in livestock farming, and public perception of veterinarians.^23–25^

We used SML combined with artificial intelligence-driven topic modeling to investigate how dog owners perceive the relationship between diet and dog health. Owner-reported health-related signs (ORHS) were defined as health-related observations described by owners in online posts as being perceived in relation to diet, rather than clinically-verified diagnoses, outcomes, or diet-specific effects (Table 1). We aimed to explore online owner perceptions of diet-related ORHS in KC and FD discussions, with FD remaining comparatively underrepresented in research relative to raw or KC feeding.

**Table 1:** Conceptual framework for interpreting owner-reported diet and health terminology in this study.

Term	Definition in this study	Examples
Owner-reported health-related signs (ORHS)	Visible or noticeable changes in a dog described by owners in online posts. These were treated as unverified owner observations, not standardized clinical measurements.	diarrhea; dull coat; scratching; weight gain; reduced appetite.
Owner interpretations/perceptions	The meaning, cause, or health relevance owners attributed to ORHS and/or diet-related experiences in online posts. Unlike ORHS, which describes what an owner observed, owner interpretations reflect how the owner explained, attributed, or connected those observations to a diet, ingredient, health condition, or other factor. These patterns were identified through topic modeling, and were treated as owner perceptions, not evidence of causality.	Diet caused diarrhea; chicken caused itching; scratching was due to seasonal allergies; changing food helped weight management.
Clinical diagnosis	A diagnosis made by a veterinarian using clinical history, physical examination, diagnostic testing, and/or an appropriate dietary trial where indicated. Clinical diagnoses were not assessed or verified in this study; any diagnoses mentioned in posts were treated as unverified owner-reported diagnoses.	Food allergy; food-responsive enteropathy; pancreatitis; atopic dermatitis; obesity.

## Materials and methods

Our observational, retrospective SML study analyzed online owner perceptions of diet-related ORHS in dogs using publicly available, open-source social media posts. The University of Surrey Ethics Committee granted a favorable ethical opinion (ref: 2802-amend 4), and informed consent was waived.

### Data collection and preprocessing

Posts discussing KC and FD, dated January 1, 2025 to July 3, 2025, were extracted from blogs, forums, Reddit, reviews, and X using Pulsar Platform™, a licensed SML tool.^26^ No private or closed-group content was included. Posts were restricted to Great Britain and Northern Ireland, written in English, and required to match predefined Boolean search criteria (Figure 1 and Figures S1 and S2). Most posts originated from Reddit (KC, 96.5%; FD, 97.6%), with the remainder from other platforms (Table S1).

**Figure 1:**
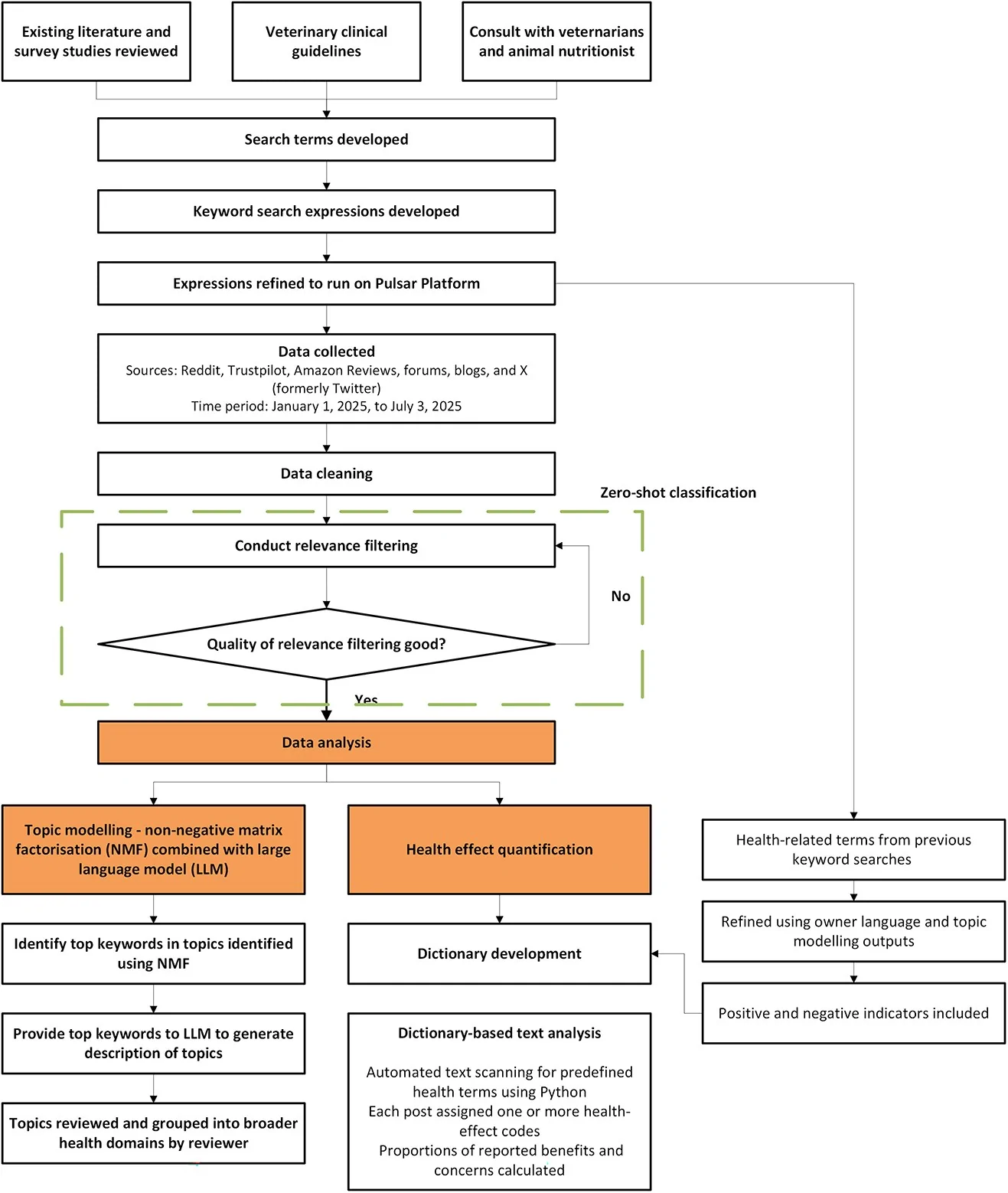
Flowchart illustrating the study methodology using social media listening and large language model–assisted analysis, including topic modeling and owner-reported health-effect quantification, to investigate dog owner perceptions of the impact of kibble/canned (KC) and fresh diets (FD) on dog health using publicly available online social media posts.

Keyword search terms were developed using published literature and veterinary clinical guidelines in consultation with 2 Royal College of Veterinary Surgeons-registered veterinarians and an Association for Nutrition-registered nutritionist.^7^^,^^9–12^^,^^27^^,^^28^ Boolean search strings were iteratively refined by 2 reviewers (Figure 2 and Figure S1 and S2). Kibble/canned diet and FD posts were analyzed independently.

**Figure 2:**
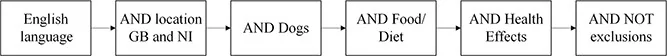
Logical structure of the Boolean search expression used to identify social media posts discussing diet and dog health. The search strategy combined 5 inclusion blocks linked by Boolean AND operators, ensuring that all key topic areas were represented within the retrieved posts. The 6th exclusion block, connected via AND NOT, was applied to remove irrelevant content such as advertisements, non-dietary discussions, or posts concerning non-dog species. Each block comprises multiple related terms, and synonyms joined with OR operators (eg, in the dog block, dog OR dogs OR puppy; in the food/diet block, dry food OR kibble OR dog meal; and in the health effects block, itch OR poo OR stomach). The overall structure was designed to maximize retrieval of relevant posts while maintaining specificity to the study scope. Abbreviations: GB = Great Britain; NI = Northern Ireland.

The FD posts were defined as those referencing fresh, human-grade, gently cooked, mildly cooked, or home-cooked diets, and therefore represented heterogeneous feeding practices. Given variability in owner terminology and overlap with raw feeding and multiple diet types, these posts were retained to capture emergent themes without loss of qualitative depth.

After preprocessing and anonymization, GPT-4o-mini (OpenAI) was used for zero-shot classification of extracted posts as relevant or irrelevant using a fixed prompt without task-specific training (Table 2).^23^^,^^24^ Relevant posts were retained for subsequent analysis.

**Table 2:** Data processing and relevance filtering results for the kibble/canned (KC) and fresh diet (FD) dog owner posts.

Diet	Initial posts (*n*)	Posts after cleaning (*n*)	Relevant posts included in analysis, *n* (%)^a^
Kibble/canned	21 896	11 000	8040 (36.7)
Fresh	2336	1435	924 (39.6)

a Relevant posts were identified following preprocessing (including language and character-length filtering) and relevance filtering using zero-shot classification. Percentages are calculated relative to the number of posts initially scraped for the dataset (eg, KC: 8040/21 896 = 36.7%).

### Topic modeling

Non-negative matrix factorization (NMF) with large language model (LLM)-assisted interpretation (GPT-4o-mini, OpenAI) was used to identify recurrent themes in owner discourse. Model selection balanced quantitative metrics, including coherence scores (Cv) and model stability, with manual review to identify parsimonious and interpretable topic structures.^23^^,^^29^^,^^30^ The LLM-generated outputs were manually reviewed against source posts and topic terms (Figure S8).

### Exploratory ORHS quantification

A rule-based text-matching dictionary was developed in Python to quantify ORHS.^31^ Terms were derived from the health-effects Boolean search block, NMF outputs, and manual post review, and grouped into non-mutually exclusive ORHS categories (Figure S9). Posts could be assigned to multiple categories but were counted once per category as concern, benefit, or both. The ORHS classifications represent owner language, not clinically-confirmed diagnoses or disease prevalence.

Further methodological detail, including exploratory sentiment classification and validation of the zero-shot classifier, NMF model selection, and exploratory ORHS quantification, is provided in the Appendix.^32^^,^^33^

## Results

All findings, including mentions of health-related terms within themes, should be interpreted according to the terminology framework presented in Table 1. Terms used in posts and reflected in the corresponding topic modeling analysis, such as “allergy,” “intolerance,” “food sensitivity,” and “pancreatitis,” represented unverified ORHS or owner perceptions rather than clinically verified diagnoses.

The KC and FD posts were analyzed separately and interpreted descriptively as patterns in discourse rather than as directly comparable diet-exposure groups, given differences in dataset size, search-string capture, potential owner motivation, and the heterogeneous terminology used to describe FD.

### Topic modeling outputs

Non-negative matrix factorization topic modeling with LLM-assisted interpretation was performed on KC (*n* = 8,040) and FD (*n* = 924) posts to understand online owner discussions surrounding perceptions of diet-related ORHS.

Intercoder reliability for the LLM model validation using the ground-truth dataset (*n* = 200 KC and 141 FD) was substantial across KC and FD posts, with agreement > 85% and Cohen’s κ = 0.72-0.80 (Table S2). The LLM model demonstrated high performance (accuracy, 0.88-0.90; F1 score, 0.91-0.92), with consistent performance during single-reviewer quality control checks (accuracy, 0.86-0.95; F1 score, 0.85-0.97; Table S3).

Topic modeling identified related patterns of owner discourse within KC and FD posts, with owners framing diet as a perceived cause, management strategy and preventive approach for dermatologic, allergy, and GI ORHS. Visible health signs, including pruritus, otitis, diarrhea, vomiting and “sensitive stomach,” were frequently reported, alongside owner descriptions of repeated trial-and-error dietary transitions involving novel proteins, elimination strategies, therapeutic diets, and alternative fresh or homemade diets.

### KC owner posts topic modeling

Topic modeling for KC posts (*n* = 8040) identified 7 manually-reviewed topics (Cv = 0.58). Representative quotes and exploratory sentiment analysis are provided in Table 3 and Figure S10A.

**Table 3:** Topic modeling results derived from NMF and LLM-assisted interpretation for kibble/canned owner posts (*n* = 8040).

Theme	Theme title	Exemplar quotes^a^	Weight, *n* (%)^b^
1	Allergies and dermatological health	“If cytopoint dropped his itch to 0/10, the origin of the itch is atopy (environmental allergies)… if you wish to make a diet switch anyway…” “We had diarrhea and skin issues… after testing, food-wise he’s allergic to several ingredients… we switched to a limited ingredient diet …” “Change the diet… Shar pei are prone to allergies… feed salmon and sweet potato… Allergies can cause ear problems… Chicken is usually the worst for allergies.”	2636 (32.8)
2	Challenges of dog dietary preferences	“How do I get my dog to eat her kibble?… add healthy topper to make it smell more like food. We’ve done sardines, pumpkin, yogurt, cheese, green beans, or no sodium chicken broth.” “…he is the pickiest eater… doesn’t enjoy any kibble, wet or raw food, I’ve had to start making him omelettes…” “picky eater … don’t mix anything. Leave a small bowl of dry food out and feed a separate bowl with a little wet dog food out at meal times.”	1444 (18.0)
3	Weight management and obesity prevention	“To lose weight … determine how many calories he needs per day… then feed an amount that will allow loss of no more than 2% body weight a week.” “We measured food in the morning and evening… severely limited table scraps… switched to lower-calorie food and her dog lost 4 pounds over the winter.” “We cut out 1/3 of his daily kibble and replaced it with green beans… hydrotherapy… lower calorie treats… eventually it all came off.”	1050 (13.1)
4	Novel protein and alternative diets for allergy management	“He can’t have poultry… does great on venison and rabbit… Been dealing with a dog who’s allergic to almost everything for 9 years.” “We did a 10-week… hydrolyzed food… introduced chicken back… symptoms returned. Now he is on a limited ingredient… doing amazingly.” “Our pup had chronic diarrhea… allergic to chicken, beef, and salmon…Hydrolyzed Protein prescription food was a game changer… good as gold ever since.”	1008 (12.5)
5	Gastrointestinal signs and perceived food responsiveness	“ …diagnosed with pancreatitis …had reoccurring stomach issues. The vet put him on prescription diet, and he’s been doing great since.” “ …has a really sensitive stomach, always getting diarrhea when he was a puppy until his vet recommends a probiotics powder that goes on his food daily.” “After trying raw and wet food styles and her still proceeding to gas… we started using pumpkin powder …slightly improved things but still pretty bad”	969 (12.1)
6	Grain-free diets and perceived cardiovascular risk	“Vets have found that dogs fed a grain free diet usually end up having major heart problems from it.” “A month on and off grains caused her to go into heart failure. Please be smarter than me!” “I’m not all that concerned about grain or grain free for my dog but she was getting these bumps on her back.”	535 (6.7)
7	Whole food and homemade feeding preferences	“Dog refuses to eat dog food (soft and dry) but will eat chicken.” “Our girl will eat kibble sometimes, but her main diet is wet dog food… sometimes gets stuff like boiled chicken breast, cooked ground beef and unseasoned salmon.” “I make them a mixture of human-grade dry dog food and cooked ground beef… They love it.”	398 (5.00)

Themes identified using non-negative matrix factorization (NMF), with large language model-assisted summarization of topic descriptors. Exemplar quotes illustrate representative posts within each theme. Theme weight (*n*, %) represents the number and proportion of posts assigned to each dominant topic.

a Quotes represent example excerpts from posts within each dataset that correspond to the identified themes and have been minimally revised to remove any identifiable information.

b Weights represent the number and proportion of posts for which each topic was identified as the dominant topic based on NMF scoring. Percentages are calculated relative to the total number of posts included in the topic modeling analysis after preprocessing and relevance filtering, and therefore sum to 100% within each dataset.

#### Theme 1. Allergies and dermatologic health

Posts in this theme discussed management of allergic and dermatologic conditions through dietary modification, with diet frequently cited as both the perceived cause and potential management approach for health concerns. Frequently reported owner concerns included pruritus, cutaneous flare-ups, recurrent otitis, yeast infections, and concurrent GI sensitivities.

Owners typically described a management strategy of sequential dietary transitions followed by periods of clinical sign monitoring. Narratives highlighted partial improvement or relapse of clinical signs, which prompted further experimentation with various brands and alternative formulations. These included veterinary-recommended hypoallergenic or prescription diets, as well as raw, fresh, or homemade options. Many owners sought advice from online communities, with few referencing veterinary consultations.

#### Theme 2. Challenges of dog dietary preferences

This theme centered on the various methods trialed for overcoming food refusal and inconsistent dietary intake. Owners frequently shared strategies to manage perceived “picky” eating behavior, including the use of toppers, texture modification, and meal enhancement to improve palatability and intake.

Some owners expressed preference for “less processed” diets, including fresh, homemade diets, food for humans, or premium formulations, alongside concerns regarding processing of kibble and the nutritional adequacy of alternative diets (eg, “Changing my dog’s food to a less processed kibble helped reduce the frequency and smelliness of her eye discharge”). Discussions also included owner questions about whether food refusal was behavioral or secondary to GI sensitivities or chronic diseases.

#### Theme 3. Weight management and obesity prevention

This theme captured posts focused on the role of diet in safe, pragmatic weight management, and obesity prevention. Posts included both personal accounts of weight-loss strategies and peer-to-peer advice, as well as owners actively seeking guidance on effective feeding approaches. Owners described implementing calorie restriction and portion control, transitioning to lower calorie or weight management formulations, feeding veterinary-recommended diets, and incorporating exercise to promote weight loss and maintain healthy body condition.

#### Theme 4. Novel protein and alternative diets for allergy management

Posts within this theme described the use of novel or alternative protein sources (eg, lamb, turkey, salmon) for perceived allergy management. Reported health concerns included pruritus, skin inflammation, recurrent otitis, and GI disturbances (eg, loose feces, vomiting, flatulence). Discussions referenced both veterinary-prescribed therapeutic diets (eg, hydrolyzed or limited ingredient formulations) and owner-initiated homemade feeding approaches, often adopted to allow more ingredient control, particularly in relation to suspected chicken allergy.

#### Theme 5. GI signs and perceived diet response

Owner posts in this theme frequently framed diet as both the perceived cause and management approach for GI health, with “sensitive stomach,” diarrhea, vomiting, flatulence, and loose feces among the most common concerns. Many posts referenced suspected food allergies (often chicken), pancreatitis, Giardia, inflammatory bowel disease, and protein-losing enteropathy. Dermatologic signs (such as sensitive skin, coat issues, and itching) were described concurrently with GI symptoms.

Owners linked fluctuations in clinical signs to specific foods or formulations and described repeated trial-and-error dietary changes involving protein substitution, specialized “sensitive stomach” diets, and additional supplements such as probiotics or pumpkin. Although some owners reported improvement after dietary modification, others described relapse or persistent health concerns despite multiple diet changes.

#### Theme 6. Grain-free diets and perceived cardiovascular risk

Discussions in this theme centered on grain-free diets and perceived associations with dilated cardiomyopathy (DCM) and heart disease. Many posts expressed concerns about potential cardiovascular risk, referencing “specialist” and “studies” linking grain-free diets to DCM, whereas others questioned the strength and validity of the evidence.

Some owners described implementing grain-free diets in response to perceived allergies whereas others shared uncertainty about safety and emphasized the importance of veterinary consultation before initiating or continuing grain-free feeding.

#### Theme 7. Whole food and homemade feeding preferences

Posts in this theme captured discussions about owner preference and dog acceptance of whole foods, such as chicken or vegetables (eg, boiled vegetables, chicken broth, egg, rice) rather than KC diets, especially when dogs refused processed food. This theme reflected owner prioritization of fresh and flavorful options that increase palatability, alongside concerns about nutritional quality. Owners discussed the nutritional value and research evidence surrounding cooked, homemade and processed diets, highlighting perceived benefits and drawbacks.

### FD owner posts topic modeling

Topic modeling for FD posts (*n* = 924) identified 6 manually reviewed topics (Cv = 0.44). Representative quotes and exploratory sentiment analysis are provided in Table 4 and Figure S10B.

**Table 4:** Topic modeling results derived from NMF and LLM-assisted interpretation for fresh diets (FD) owner posts (*n* = 924).

Theme	Theme title	Exemplar quotes^a^	Weight, *n* (%)^b^
1	Perceptions of raw and fresh feeding on health	“I have been using a fresh diet for a number of months … really seen a difference in his coat and general demeanour.” “ …raw and freshly cooked food helps dogs live healthier longer lives …” “ …life changing dog transformations from switching to a whole food, complete raw diet.”	156 (16.9)
2	Challenges of dog dietary preferences	“tried raw … rejected wet … now turning her nose up at dry … I’ve tried toppers, eggs, fish oil, toys, games, slow feeders …” “Both are impossibly picky … try different kibble … there are fresh cooked brands … I mix it with kibble … this changed her health and energy significantly.” “We used to mix fresh/frozen dog food with kibble … then boiled chicken with kibble … something works for a while, then she gets sick of it and we have to change.”	195 (21.1)
3	Preference for human-grade diets	“The only thing that helped my itchy bull terrier was switching to human grade food …” “Feeding human grade salmon, rice, sweet potato and diced veggies … cleared his stomach and skin issues.” “ …try the fresh/human grade dog food — it’s healthier than kibble.”	98 (10.6)
4	Homemade diets for allergy and gastrointestinal management	“I started making food at home. Her constant stank fart’s disappeared … lot less scratching.” “I think the human grade fresh chicken is better, he doesn’t itch from it …they are eating better than some people” “I make a big pot per week …can’t imagine ever feeding dry dog food. It’s more expensive, more work but they love it and a benefit is that they eat more but poop less (think the new food has a high moisture content).”	159 (17.2)
5	Novel protein and therapeutic diets for dermatological and GI health	“ …switched to salmon formula, and there are no eye stains, no tears at all.” “After many upset-stomach episodes and emergencies … appetite, skin, and stools improved after switching to veterinary formulated diet.” “ …when he’d have grains, he’d get really itchy, drool a lot, lick his paws and was incredibly prone to ear infections … he’s old now… stick to food directly from our vet.”	174 (18.8)
6	Perceived health benefits and risks of fresh diets	“There is at least one metric by which fresh is better: hydration.” “ …fresh pet foods … can lead to pancreatitis in dogs due to high fat content …” “ …fresh food brands can have way too high of a fat content …not truly balanced nutrition …”	142 (15.4)

Themes identified using non-negative matrix factorization (NMF), with large language model-assisted summarization of topic descriptors. Exemplar quotes illustrate representative posts within each theme. Theme weight (n, %) represents the number and proportion of posts assigned to each dominant topic.

a Quotes represent example excerpts from posts within each dataset that correspond to the identified themes and have been minimally revised to remove any identifiable information.

b Weights represent the number and proportion of posts for which each topic was identified as the dominant topic based on NMF scoring. Percentages are calculated relative to the total number of posts included in the topic modeling analysis after preprocessing and relevance filtering and therefore sum to 100% within each dataset.

#### Theme 1. Perceptions of raw and fresh feeding on health

This theme centered on owner beliefs that raw and “gently cooked” FD are healthier alternatives to “processed kibble” (eg, “I said it [raw food] was less processed, especially compared with kibble cooked at very high temperatures”). Many owners described improvements after dietary change, including improved GI function, skin, and coat condition, energy level, and overall vitality. Owner discussions framed raw and FD as preventative nutrition, linking them to long-term well-being and lower perceived risk of GI, dermatologic, and chronic health problems.

#### Theme 2. Challenges of dog dietary preferences

Posts in this theme described owner challenges with dogs perceived as “picky” eaters. Owners reported repeated experimentation with fresh, human-grade diets, switching brands, adding textures, combining wet and dry foods, and supplementing with homemade food. They also expressed concerns regarding adequate nutrition because of frequent diet changes and inconsistent eating patterns.

#### Theme 3. Preference for human-grade diets

This theme captured owner posts advocating for “human-grade” diets and discussing their perceived advantages over KC diets. These diets were described as higher quality, more “natural” and “less processed” than kibble diets. Owners reported improvements after dietary transitions in dogs with suspected sensitivities or allergies, including resolution of GI upset, decreased pruritus, improved coat condition, and increased appetite.

#### Theme 4. Homemade diets for allergy and GI management

Posts in this theme centered on owners preparing home-cooked meals in response to GI or allergy-related concerns. Owners discussed ingredient quality and tailoring diets to their individual dogs’ needs, and reported benefits including improved fecal consistency, decreased scratching, decreased flatulence, and enhanced coat quality. However, many posts expressed concerns regarding the nutritional completeness of homemade diets, with some owners referencing veterinary nutritionist guidance for balanced formulation.

#### Theme 5. Novel protein and therapeutic diets for dermatologic and GI health

This theme focused on owner discussions of dietary management for suspected food sensitivities, skin concerns, and GI concerns, including chronic diarrhea, vomiting, pruritus, otitis, and “sensitive stomachs.” Owners described switching among commercial diet formulations, including protein-elimination strategies, limited ingredient diets, and veterinary-recommended diets, such as hydrolyzed or prescription diets. Although some owners reported improvement in ORHS after dietary change, others described persistent flare-ups and difficulty identifying consistently tolerable options.

#### Theme 6. Perceived health benefits and risks of fresh diets

This theme reflected mixed and polarized owner perspectives on FD compared with KC diets. Owners described perceived benefits, including improved hydration, energy, and digestive function, and adverse outcomes including diarrhea, vomiting, weight gain, and owner-reported pancreatitis, often attributed to the diet’s high fat content. Discussions questioned the supporting evidence base, regulatory definitions, formulation standards, and extent of available veterinary guidance.

### Exploratory ORHS quantification

The exploratory text-based dictionary ORHS classifier showed high overall performance for detection of any ORHS category in the manually annotated subset (*n* = 100; precision, 0.95; 95% CI, 0.91-0.98; recall, 0.97; 95% CI, 0.92-0.98; F1 score, 0.96) and all evaluable individual category F1 scores ≥0.75 (Table S4). Additional evaluation of script-positive posts from the KC and FD datasets (*n* = 547) showed an overall positive predictive value (PPV) of 0.91 (95% CI, 0.89-0.93) and false-positive rate of 0.09 (95% CI, 0.07-0.11), with category-specific concern PPV ranging from 0.83-1.00 and benefit PPV ranging from 0.46-1.00 (Table S5).

The most frequently identified ORHS categories, which were non-mutually exclusive, within the KC (*n* = 8040) dataset were GI (28.4%), allergy/intolerance (24.0%), appetite/fussy eating (13.8%), skin/coat (11.4%), and general health/illness (9.5%; Figure 3 and Tables S6 and S7). In the FD dataset (*n* = 924), similar ORHS ranking patterns were observed, with the most frequently identified categories being GI (30.5%), allergy/intolerance (21.0%), appetite/fussy eating (14.4%), general health/illness (13.5%), and skin/coat (12.3%).

**Figure 3:**
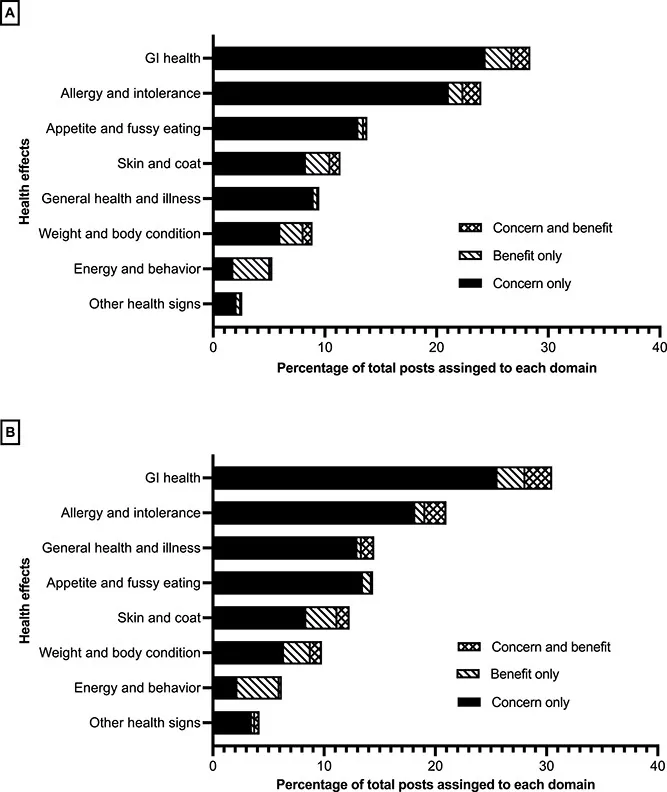
Distribution of posts assigned to owner-reported health effects in (A) kibble/canned (KC) and (B) fresh diet (FD) social media datasets (*n* = 8040 and 924, respectively). Stacked bar charts show the percentage of total posts classified into each health effect and polarity (concern, benefit, or both) using a predefined text-based dictionary implemented in Python. Posts could be assigned to multiple health effects; within each health effect, each post was counted once as a concern, benefit, or both. Thus, totals represent health effect level post counts rather than individual mentions. For example, in panel (A), owner posts classified as mentioning gastrointestinal (GI) health: concern only 1961/8040 (24.4%), benefit only 196/8040 (2.4%), or both 129/8040 (1.6%), while the remaining posts did not mention the GI category but may have mentioned other category health terms. “Other health signs” include ear, ocular, oral, and dental-related terms.

Co-occurrence analysis demonstrated consistent clustering of ORHS within both KC and FD datasets (Figure 4). Skin/coat mentions most frequently co-occurred with allergy/intolerance mentions within the same owner posts (KC 52.1%; FD 50.0%), whereas allergy/intolerance mentions also commonly co-occurred with GI mentions (KC 24.6%; FD 35.1%). Among posts mentioning appetite/fussy eating, GI mentions co-occurred in 22.3% of KC posts and 32.3% of FD posts. Among posts mentioning general health/illness, GI mentions co-occurred in 23.6% of KC posts and 29.6% of FD posts. Overall, these post-level patterns indicate that owners commonly described multiple concurrent ORHS, particularly across GI, dermatologic, allergy-related, and appetite-related categories.

**Figure 4:**
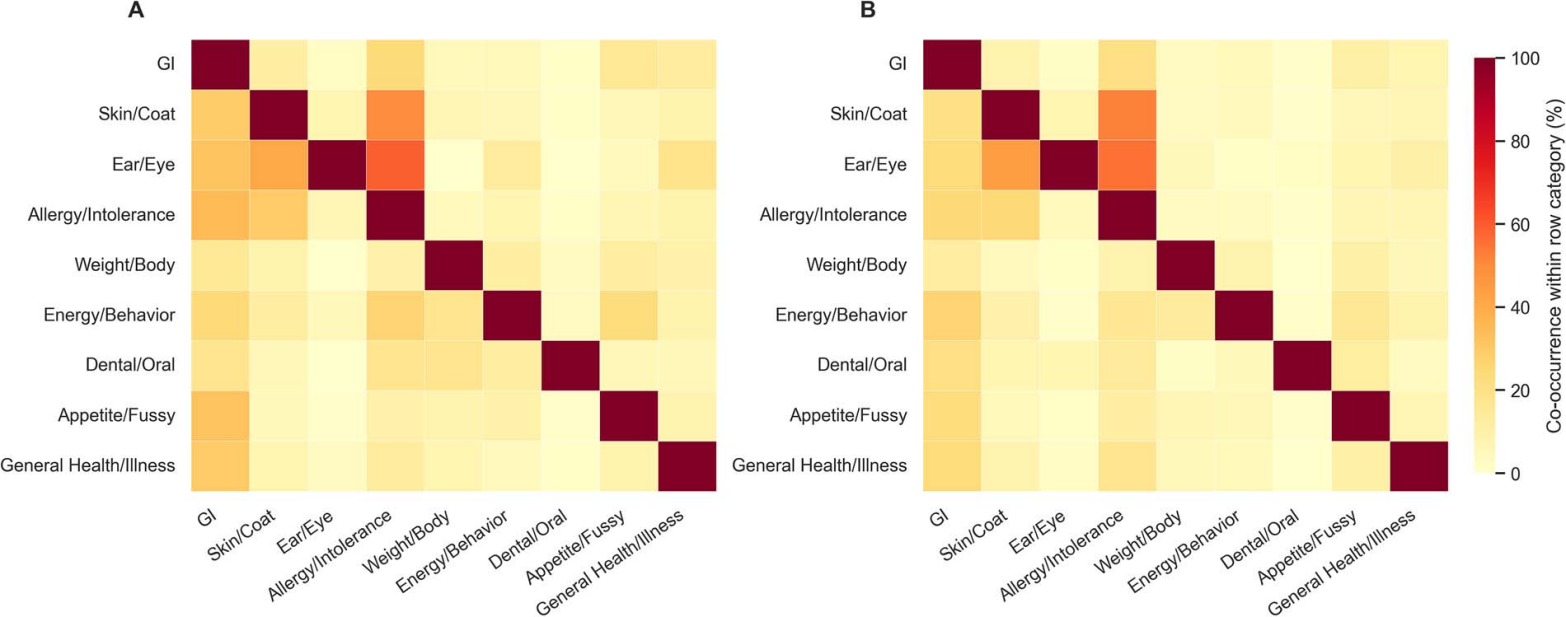
Row-normalized heatmap showing post-level concurrence of owner-reported observable health-related signs in (A) kibble/canned (KC) and (B) fresh diet (FD) social media datasets (*n* = 8040 and 924, respectively). Each cell represents the percentage of owner posts containing the row category that also mentions the column category, calculated using an exploratory text-based dictionary classifier developed in this study. Posts could be assigned to multiple ORHS categories but were counted once per category as concern (eg, diarrhea), benefit (eg, shiny coat), or both, representing post-level classifications within each dataset of posts (KC or FD) and not clinically confirmed diagnoses or disease prevalence. Darker cells indicate greater concurrence of owner-reported health-related signs. “Other health signs” include ear, ocular, oral, and dental-related terms. Abbreviation: GI = gastrointestinal.

## Discussion

Our study applied SML with LLM-assisted topic modeling to examine naturally-occurring, unsolicited owner narratives of diet-related ORHS in dogs across KC and FD online discourse. We identified dominant visible ORHS, including GI, dermatologic, and weight-related mentions. Owners used observable signs, such as fecal consistency, vomiting, flatulence, pruritus, coat quality, body weight, and appetite, as pragmatic health indicators to guide dietary transitions. Because the KC and FD datasets differed in sample size, search string capture, unverified diet classification, and diet category heterogeneity, they were analyzed separately and not interpreted as comparable dietary exposure groups. Nevertheless, similar broad patterns were observed within each dataset. By identifying visible ORHS that owners appeared to use when making diet-related decisions, our study may help veterinarians anticipate common owner concerns, guide evidence-informed nutrition discussions, and support informed dietary decision-making.

Topic modeling, exploratory ORHS quantification, and co-occurrence analysis indicated that owners framed diet as both a perceived cause of and management strategy for ORHS, with multiple signs commonly discussed within individual posts, particularly allergy/intolerance with skin/coat signs and GI with allergy-related signs (Figure 4). This owner co-reporting overlaps with clinical presentations in which GI and dermatologic signs may occur concurrently, such as food-responsive enteropathy and cutaneous adverse food reactions.^34^^,^^35^ Although these owner-reported patterns cannot establish clinical associations, temporality, shared pathophysiology, or diet-specific effects, they identify ORHS clusters that may be clinically relevant and warrant further investigation of potential biological links between GI and dermatologic disease in the context of diet. Clinical studies are needed to determine whether the identified ORHS, such as scratching, loose feces, and lethargy, correspond to validated diagnoses, objective biomarkers, or measurable biological differences across defined dietary exposures.

Similarly, survey-based studies examining owner motivations, particularly for raw and grain-free diets, have consistently reported digestive disturbance, fecal quality, coat condition, and diet-related allergy or intolerance as among the most frequently cited factors influencing dietary change.^10–12^^,^^36^ However, these studies have relied on structured questionnaires administered to predefined participant groups, and recruitment strategies may over-represent owners already feeding the diet in question (eg, raw diets) or veterinary professionals, thereby limiting the generalizability of findings to the wider dog-owning population.^11^

In contrast, we utilized social media platforms to decrease researcher prompting and selection bias, capturing a large volume of unsolicited owner discussions across a broader range of diet types, including FD, and providing more naturalistic insight into owner perceptions and beliefs. Despite these advantages, self-reported online data remain susceptible to self-selection and concern-driven posting biases. Although SML captures unsolicited owner experiences at scale, individuals with negative, extreme, or salient experiences may be more motivated to contribute and may therefore disproportionately shape the discourse captured, a limitation shared by SML, surveys, interviews, and other qualitative research methods.^37–39^ Therefore, our findings should be interpreted as reflecting the concerns and experiences expressed by posting owners, rather than the prevalence of these experiences in the wider dog-owning population, limiting generalizability.

Across several themes, owners used terms such as “human-grade,” “natural,” “fresh,” and “less processed” when discussing diet quality, perceived health value, and dietary decision-making (KC theme 7; FD themes 1 and 3). These descriptors may reflect consumer-facing marketing language, personal diet and health values, or peer discussion, and indicate that owners’ perceptions of dog food quality often are shaped by subjective interpretations rather than standardized nutritional definitions.^17^ Similar descriptors in food research in humans are associated with perceived quality, naturalness, healthfulness, and acceptability, guiding food choices based on consumer perceptions rather than validated nutritional, regulatory, or evidence-based clinical recommendations.^17^^,^^40–42^

Consistent with this concern, the use of such language in dog owner discussions suggests an anthropomorphic transfer of nutritional values of humans, particularly the prioritization of naturalness, minimal processing, and perceived healthfulness.^43–45^ Previous dog owner survey research has shown that owners’ personal dietary patterns and beliefs (eg, vegan, vegetarian, sustainability-focused, or grain-free preferences) are strong predictors of the feeding practices adopted for their pets.^7^^,^^11^^,^^44^^,^^46^ Together, these findings suggest that dog dietary decision-making is not only influenced by perceived product attributes, but also by increasing awareness of the relationships among diet, health, disease prevention, and longevity.^22^^,^^47^^,^^48^

Our study had some limitations. Although promotional content and duplicate posts were excluded where identified, residual commercial influence, brand advocacy, reposting, or influencer-related content cannot be fully excluded. In addition, despite a multi-platform search strategy, > 95% of posts originated from Reddit, introducing potential platform-specific biases, including amplification of highly engaged posts and possible repeated contributions from the same users across subreddits. Demographic information for owners and dogs was largely unavailable, and all data were based on owner-reported observations and perceptions rather than clinical examination or verified diagnoses. Variability in owner language may have limited consistent capture and interpretation of ORHS in the exploratory ORHS quantification, because owners often describe signs informally, indirectly, or ambiguously, and the same term may carry different meanings depending on context.

Dietary exposure could not be standardized or confirmed because posts often used inconsistent terminology, referred to more than one diet type, or described mixed feeding practices. Therefore, the observed post-level narratives and ORHS co-occurrence patterns cannot be attributed to defined nutritional exposures or interpreted as evidence of diet-specific health effects. The FD category was particularly heterogeneous, including posts referring to fresh, human-grade, gently cooked, home-cooked, and potentially overlapping raw or mixed feeding practices. Although the FD topic model demonstrated high stability and acceptable interpretability, its lower coherence compared with the KC model suggests weaker topic separation and more thematic overlap. Therefore, FD-derived topic interpretations should be interpreted cautiously. Finally, the dictionary-based ORHS quantification should be interpreted as exploratory because it was designed to summarize post-level patterns in owner language rather than provide a fully validated classification model.

Overall, our findings should be interpreted as reflecting online owner perceptions and providing insights into how owners discuss diet-related discourse, rather than representing clinically validated health outcomes or confirmed diet-related effects.

## Conclusions

Our study provides a large-scale SML and LLM-assisted topic modeling analysis of online owner perceptions of diet-related ORHS in dogs. Owners frequently associated diet with GI, dermatologic, and allergy/intolerance-related signs, using visible ORHS such as fecal quality, skin condition, and appetite to judge perceived dietary success. Our study provides veterinarians with a large-scale, descriptive summary of online owner-reported perceptions and highlights common concerns that may arise during veterinary nutrition consultations. Future studies should investigate whether measurable physiological, biochemical, or clinically validated outcomes correlate with the identified ORHS that clients use to assess diet and dog health, thereby supporting informed dietary recommendations and ultimately enhancing the health and welfare of companion animals.

## Supplementary Material

Supplementary_caption_aalag245(1)

## Data Availability

The data supporting the findings of this study are included in the manuscript and its supplementary material. Where ethically feasible, de-identified data and analysis code will be made publicly available via a GitHub repository upon publication (https://github.com/jkaur2025/Social-Listening-Data.git). MS Presidio was used to remove all personally identifiable information before data sharing.

## Off-label antimicrobial declaration

Authors declare no off-label use of antimicrobials.

## Institutional animal care and use committee or other approval declaration

Approved by the University of Surrey Ethics Committee (Ref: 2802—amend 4). Written informed consent was waived because consent for participation was not required in accordance with national legislation and institutional requirements.

## Appendix

### Methods

#### Data collection and preprocessing

Post inclusion criteria were restricted to English-language posts returned by the predefined Boolean search expressions and geographically restricted in Pulsar Platform™ to Great Britain and Northern Ireland. Posts containing languages other than English were excluded. Although geographical filtering was applied during data extraction, user-reported location metadata was unavailable for > 97% of posts, and demographic data (country, gender) were unavailable for > 95% of posts. Posting volume was broadly consistent, supporting temporal stability, with a notable increase in FD dataset posts in June 2025 (Figure S3). Most posts originated from Reddit (KC: 21 130/21 896, 96.5%; FD: 2279/2336, 97.6%), with the remaining posts from other platforms (Table S1).

The Boolean search expressions were iteratively refined through multiple rounds to maximize sensitivity and ensure compatibility with the technical parameters of the Pulsar Platform™ (query length and operator limits), prioritizing broad capture of relevant posts rather than restrictive specificity (Figures S1 and S2).

Posts were preprocessed in Python (3.12.7; pandas 2.3.1) using standard text-cleaning procedures (lowercasing, white space normalization, removal of URLs, usernames, special characters, duplicate and near-duplicate entries). Titles were concatenated with content. Character-length filtering thresholds were defined using a pilot subset (KC, *n* = 9924; FD, *n* = 2109) informed by distribution plots and manual checks, then applied and confirmed in the full dataset (KC, 100-1300; FD, 50-1700 characters; Figures S4 and S5). Posts were not mutually exclusive.

Advertisements, product reviews, and promotional posts were filtered using zero-shot relevance filtering. To decrease duplication, reshares, reposts, and repeated or near-identical content, iterative fuzzy matching in Python was used to identify posts with > 90% text similarity. Similar posts were removed, and the process was repeated until no further overlap was observed. However, residual promotional and influencer content may not be fully excluded.

#### Zero-shot classification

Zero-shot classification was performed using GPT-4o mini (OpenAI; accessed September 2025 to February 2026) via the OpenAI API. The model was used to classify each social media post as either relevant or irrelevant based on predefined inclusion and exclusion criteria (full prompt in Figure S6). Posts were classified individually, with one post submitted per API call. The model was configured with a temperature setting of 0.0, max_tokens = 5, and *n* = 1 response per post. Posts referring to both diet types were included in each dataset (KC and FD) if they also described a health effect, ensuring that all relevant discussions were captured. The LLM classification prompt was iteratively refined during pilot testing to improve clarity and alignment with the coding criteria, beginning with broad definitions and adjusted after pilot testing and manual spot checks. The final prompt then was fixed for the final zero-shot classification workflow.

Zero-shot classification model performance was evaluated by comparing human and LLM classifications using standard performance measures commonly applied in machine learning, including accuracy, precision, recall, and the F1 score. These were calculated as follows:

$$ Accuracy=\frac{\mathrm{TP}+\mathrm{TN}}{\mathrm{TP}+\mathrm{TN}+\mathrm{FP}+\mathrm{FN}} $$

$$ Precision=\frac{\mathrm{TP}}{\mathrm{TP}+\mathrm{FP}} $$

$$ Recall=\frac{\mathrm{TP}}{\mathrm{TP}+\mathrm{FN}} $$

$$ F1\ score=2\times \frac{\mathrm{Precision}\times \mathrm{Recall}}{\mathrm{Precision}+\mathrm{Recall}} $$

where *TP* = true positives, *TN* = true negatives, *FP* = false positives, and *FN* = false negatives.

Precision measures the model’s prediction accuracy for the relevant class, defined as the proportion of posts predicted as relevant that were truly relevant. Recall measures the model’s sensitivity, representing the proportion of all truly relevant posts that were correctly identified. The F1 score provides a balanced measure that accounts for both false positives and false negatives. All metrics were calculated treating relevant posts as the positive class.

A ground-truth dataset was created to validate model performance. Two reviewers independently coded 200 posts from the KC dataset and 141 posts from the FD dataset according to the predefined relevance criteria. Posts were classified as relevant if they (1) referred to a dog, (2) described diet or feeding practices, and (3) included a health-related observation or outcome (positive, negative, or neutral). Intercoder reliability among reviewers was assessed using Cohen’s κ to evaluate consistency in manual annotation. Discrepancies among reviewers subsequently were adjudicated by the original annotator, who had more familiarity with the dataset and study framework. The adjudicated labels were treated as the final ground-truth reference set for evaluation of GPT-4o mini zero-shot classifications.

Intercoder reliability was substantial across both datasets, with reviewer agreement exceeding 85% and Cohen’s κ values ranging from 0.72 to 0.80 (Table S2). Ground-truth validation demonstrated high model performance, which was consistent with ongoing single-reviewer quality control validation checks conducted by reviewer 1 (Table S3). The intercoder reliability and model performance metrics support that the zero-shot classification approach generalized effectively to both KC and FD owner posts in the ground-truth validation and remaining posts.

#### NMF topic modeling

Text was tokenized and lemmatized (spaCy 3.8.7), removal of stopwords (nltk) and custom terms (eg, dog-food brands; with min_df = 10; max_df = 0.95; n-grams 1-2). Candidate topic models (k = 4-15) were fitted, and the top 20 keywords from the topics were provided to the LLM to generate human-readable descriptive summaries and apply sentiment classification (“positive,” “negative,” or “neutral”; Figure S7). NMF topic models were compared using coherence metrics (Cv, UMass, and normalized pointwise mutual information [NPMI]), topic redundancy, topic uniqueness, reconstruction error, and model stability across repeated runs using 4 random seeds (42-45; gensim 4.3.3; Figure S9). Candidate solutions then were reviewed manually using top-weighted terms, exemplar posts, and topic labels to assess topic separation, interpretability, and thematic distinctiveness. Final solutions were selected by balancing quantitative metrics with review by humans, prioritizing parsimonious and interpretable topic structures.

For the FD dataset, adjacent higher-k solutions showed broadly similar quantitative performance but primarily subdivided existing themes rather than identifying substantively distinct additional topics. Therefore, the k = 6 solution was retained as the most interpretable and parsimonious representation of the FD dataset. Stability values were consistently close to 1.0 across random seeds, supporting robustness of the retained topic solutions. However, the lower FD coherence score compared with the KC model suggests weaker topic separation and higher thematic overlap; FD-derived topic interpretations therefore should be interpreted with caution.

Topic interpretation for NMF outputs was performed using GPT-4o mini (OpenAI; accessed February 20, 2026) via the OpenAI API. For each topic, the model was provided with the top 20 weighted NMF terms and up to 10 representative posts associated with that topic. Topics were processed in batches of 3 topics per API call, using the default OpenAI API temperature of 1. For each topic, the model was asked to generate a descriptive label, a 2-3 sentence summary, a topic sentiment classification (positive, negative, or neutral), an exemplar quote, and a ranked list of top example posts for review (full prompt in Figure S7). The model was prompted using a standardized prompt and JSON-formatted output structure to improve consistency across topic interpretations.

All LLM-generated topic interpretations were manually reviewed by a human investigator, and outputs were refined where necessary to ensure consistency with the underlying topic content, study framework, and contextual detail. In addition, representative posts, NMF-derived weighted terms, and top-ranked topic posts were manually reviewed alongside the LLM-generated interpretations to improve interpretive accuracy and contextual relevance of the topic summaries and descriptions before final analysis.

#### Exploratory sentiment classification

Exploratory zero-shot sentiment classification was performed using GPT-4o mini (OpenAI; accessed 02/22/26) via the OpenAI API. Text was classified into 3 predefined categories (positive, negative, neutral) regarding dog diet and health outcomes. Figure S10 shows the LLM sentiment analysis of KC and FD posts. To optimize throughput, text samples were processed in sequential batches of 20 per API call (preserving the NMF csv output file post order), with each post labeled individually within the batch and returned in JSON format. The temperature was set to 0.0 to eliminate stochastic variation and produce deterministic outputs.

#### Exploratory ORHS quantification

A rule-based dictionary was developed using the Boolean search expressions health-effects block, NMF outputs, and manual review of owner posts (Figure S11). All posts classified as relevant by the zero-shot classification were analyzed for ORHS using the rule-based dictionary. This dictionary classified health effects into 9 non-mutually exclusive ORHS categories: GI health, skin/coat, ear/eye, allergy/intolerance, weight/body condition, energy/behavior, dental/oral, appetite/fussy eating, and general health/illness. Category-specific dictionaries included both concern and benefit expressions and were designed to capture informal owner language as well as more explicit health-related sign descriptions.

Text was analyzed using the full post text, including titles where available, and contextual rules, including negation handling, were applied to decrease false positives from ambiguous terms, product names, and non-health uses of matched words. For each post, domain-level outputs were coded as binary presence/absence variables, such that repeated mentions within the same domain and polarity were counted once per post, whereas all unique matched evidence terms were retained for inspection.

Posts could be assigned to multiple ORHS categories but were counted once per category as concern (eg, diarrhea), benefit (eg, shiny coat), or both. Percentages represent ORHS post-level classifications within the separately analyzed KC and FD datasets and do not represent clinically-confirmed diagnoses or disease prevalence. Frequencies and row-normalized co-occurrence patterns then were summarized across datasets.

Classifier performance was evaluated using a manually-annotated subset (*n* = 100) by a single reviewer using precision, recall, F1 score, and PPV (Table S4). An additional evaluation was performed on script-positive posts from the KC and FD datasets (*n* = 547) to estimate category-level PPV and false-positive rate (Table S5).

## Abbreviations

CI: confidence interval
Cv: coherence score
DCM: dilated cardiomyopathy
FD: fresh diets (including human-grade, gently/mildly cooked, and home-cooked)
FN: false negatives
FP: false positives
GI: gastrointestinal
k: topics
KC: kibble/canned diets
LLM: large language model
NMF: non-negative matrix factorization
ORHS: owner-reported health-related signs
PPV: positive predictive value
SML: social media listening
