# Supplementary material for: An observational retrospective study of online owner perceptions of diet-related health signs in dogs using social media listening
Source: J Vet Intern Med. 2026 Oct 26;40(5):aalag245. doi: 10.1093/jvimsj/aalag245 (PMC13704794; doi:10.1093/jvimsj/aalag245)
Supplement: Supplementary file 1 [file supplementary_caption_aalag245(1).docx]

Figure S1. Boolean search expression for kibble/canned diet (KC) posts. Boolean search expression used to identify publicly available KC-related posts discussing owner-reported health-related signs. (A) Health concerns (B) Health benefits.

(A)

(LANG en) AND (LOCATION GB) AND (dog OR dogs OR puppy OR puppies OR pup OR pooch OR pooches OR "fur baby" OR "fur babies" OR furbaby OR furbabies OR hound OR hounds OR "my boy" OR "my girl") AND ("dog food" OR dogfood OR "pet food" OR "dog meals" OR "dog meal" OR "pet meal" OR "pet meals" OR "senior food" OR "senior diet" OR "puppy food" OR "puppy diet") AND (ultra-processed OR "ultra processed" OR "processed stuff" OR kibble OR kibbel OR kible OR kibblee OR "dry food" OR dryfood OR "dried food" OR biscuit OR biscuits OR biskit OR biskits OR bisquit OR bisquits OR food AND can OR caned OR cans OR canned OR tin OR tins OR tinned OR tined OR nut OR nuts OR nuggets OR pouch OR pouches OR tray OR trays OR Tesco OR Aldi OR Sainsbury OR Sainsburys OR Lidl OR Waitrose OR "M&S" OR asda OR morrisons OR coop OR co-op OR cooperative OR "own brand" OR supermarket OR "wet food" OR "dry mix" OR "crunchy food" OR "grain free" OR grain-free OR vegan OR vegetarian OR gourmet OR Butcher's OR Pedigree OR Friskies OR "Fit+Fun" OR MultiFit OR "Pet's Budget" OR Wagg OR Cesar OR "Hi Life" OR Sheba OR Mediterranean OR "Royal Canin" OR "Purina" OR Hills OR Eukanuba OR Advance OR IAMS OR Chappie OR "James Wellbeloved" OR "Lily's Kitchen" OR Tails OR Cannagan OR Forthglade OR Animonda OR Rocco OR Farmina OR Applaws OR "Happy Dog" OR Wolfsblut OR Orijen OR Acana OR "Almo nature" OR Yarrah OR Prins OR Harringtons OR Wainwrights OR "Wellness Core" OR Akela OR Winalot OR Skinner's OR Burns OR "Arden Grange" OR Burgess OR Bakers OR "Pooch & Mutt") AND ("health issue" OR "health issues" OR "skin issue" OR "skin issues" OR coat OR "digestive issue" OR "digestive issues" OR "low energy" OR lethargic OR lethargy OR "weight gain" OR "weight gains" OR obese OR obeisty OR obesity OR allergy OR allergys OR allergies OR allergic OR alergy OR alergys OR alergies OR alergic OR alergy OR allegy OR intolerance OR intolerances OR intollerance OR intollerances OR intolerent OR intolerant OR itch OR itchy OR itches OR itching OR itcy OR itchey OR scratching OR scratch OR scratchs OR scratches OR scratched OR scracth OR scracths OR diarrhea OR diarhea OR diahrea OR diarrea OR diarreah OR diahorrea OR diarrhoea OR diarhoea OR diarrheas OR diarrhoeas OR vomit OR vomits OR vomiting OR vomited OR vommit OR vommiting OR vommitted OR "flaky skin" OR "flakey skin" OR dermatitis OR dermititis OR "dry skin" OR infection OR infections OR infectious OR "soft feces" OR "soft faeces" OR "nasty poo" OR smelly OR fart OR farts OR farting OR farted OR faart OR faarts OR faarting OR flatulence OR flatulent OR "anal gland" OR "anal glands" OR "anel gland" OR "anel glands" OR analgland OR scoot OR scoots OR scooting OR scooted OR "back end" OR back-end OR dehydration OR dehydrated OR dehydrate OR dehydrates OR dehydrating OR "poor hydration" OR dull OR bloat OR bloats OR bloated OR bloating OR "upset stomach" OR "upset stomachs" OR "sensitive stomach" OR "sensitive stomachs" OR "sensitive tummy" OR "sensitive tummies" OR "sensitive tum" OR "upset tummy" OR "upset tummies" OR "upset tum" OR "hypoallergenic" OR "fussy eater" OR "picky eater" OR sick OR sickness OR sickly OR tarter OR tartar OR "soft stool" OR "soft stools" OR "runny poo" OR "runny poop" OR "loose poo" OR "loose poop") AND NOT RETWEET

(B)

(LANG en) AND (LOCATION GB) AND (dog OR dogs OR puppy OR puppies OR pup OR pooch OR pooches OR "fur baby" OR "fur babies" OR furbaby OR furbabies OR hound OR hounds OR "my boy" OR "my girl") AND ("dog food" OR dogfood OR "pet food" OR "dog meals" OR "dog meal" OR "pet meal" OR "pet meals" OR "senior food" OR "senior diet" OR "puppy food" OR "puppy diet") AND (ultra-processed OR "ultra processed" OR "processed stuff" OR kibble OR kibbel OR kible OR kibblee OR "dry food" OR dryfood OR "dried food" OR biscuit OR biscuits OR biskit OR biskits OR bisquit OR bisquits OR food AND can OR caned OR cans OR canned OR tin OR tins OR tinned OR tined OR nut OR nuts OR nuggets OR pouch OR pouches OR tray OR trays OR Tesco OR Aldi OR Sainsbury OR Sainsburys OR Lidl OR Waitrose OR "M&S" OR asda OR morrisons OR coop OR co-op OR cooperative OR "own brand" OR supermarket OR "wet food" OR "dry mix" OR "crunchy food" OR "grain free" OR grain-free OR vegan OR vegetarian OR gourmet OR Butcher's OR Pedigree OR Friskies OR "Fit+Fun" OR MultiFit OR "Pet's Budget" OR Wagg OR Cesar OR "Hi Life" OR Sheba OR Mediterranean OR "Royal Canin" OR "Purina" OR Hills OR Eukanuba OR Advance OR IAMS OR Chappie OR "James Wellbeloved" OR "Lily's Kitchen" OR Tails OR Cannagan OR Forthglade OR Animonda OR Rocco OR Farmina OR Applaws OR "Happy Dog" OR Wolfsblut OR Orijen OR Acana OR "Almo nature" OR Yarrah OR Prins OR Harringtons OR Wainwrights OR "Wellness Core" OR Akela OR Winalot OR Skinner's OR Burns OR "Arden Grange" OR Burgess OR Bakers OR "Pooch & Mutt") AND (improve OR improves OR improved OR improving OR improvement OR improvements OR improved OR benefit OR benefits OR benefited OR benefitted OR benefiting OR benefitting OR better OR digest OR digests OR digesting OR digested OR digestion OR "gut health" OR firm OR firmer OR firming OR "more normal" OR "easier to pick up" OR healthier OR "less fart" OR "less farts" OR "less farting" OR "less flatulence" OR shinier OR shiny OR shininess OR gloss OR glossy OR glossiness OR soft OR softer OR softest OR softness OR "clear skin" OR itch OR itching OR itchy OR itches OR "less scratching" OR "reduced allergy" OR "reduced allergies" OR hypoallergen OR hypoallergenic OR hypoallergy OR hypoalergic OR energy OR energies OR energetic OR energetically OR vitality OR vital OR weight OR weight OR slim OR slimmer OR slimmest OR slimming OR "more active" OR playful OR playfully OR playfulness OR playfull OR activity OR activities OR calm OR calming OR calmer OR calmest OR calmness OR live OR lives OR lived OR living OR livelier OR liveliest OR happier OR happiness OR thriving OR thrive OR thrives OR thrived OR hydrate OR hydrates OR hydrated OR hydrating OR hydration OR appetite OR appetites OR appetitive OR appetit OR appetizing OR appetising OR apeti OR "less fussy" OR "less picky" OR breath OR breaths OR breathing OR breathe OR tummy OR tummies OR stomach OR sensitive OR poo OR poos OR stool OR stools OR waste OR coat OR "licking the bowl" OR "licks the bowl" OR "bowl clean") AND NOT (survey OR participant OR participants OR recruitment OR transgender OR trans OR interview OR "google form") AND NOT RETWEET

Figure S2. Boolean search expression for fresh diets (FD) posts. Boolean search expression used to identify publicly available FD-related posts discussing owner-reported health-related signs. FD included human-grade, gently or mildly cooked, and home-cooked diets. (A) Health concerns (B) Health benefits.

(A)

(LANG en) AND (LOCATION GB) AND (dog OR dogs OR puppy OR puppies OR pup OR pooch OR pooches OR "fur baby" OR "fur babies" OR furbaby OR furbabies OR hound OR hounds OR "my boy" OR "my girl") AND ("dog food" OR dogfood OR "pet food" OR "dog meals" OR "dog meal" OR "pet meal" OR "pet meals" OR "senior food" OR "senior diet" OR "puppy food" OR "puppy diet") AND ("fresh food" OR "fresh dog food" OR "fresh pet food" OR "fresh diet" OR "minimally processed" OR "minimally-processed" OR "human-grade" OR "human grade" OR "human quality" OR "gently cooked" OR "frozen dog" OR " frozen pet" OR "cold pressed" OR "refrigerated dog food" OR "refrigerated pet food" OR "refrigerated diet" OR "natural diet" OR "natural dog food" OR "natural pet food" OR "freshly made pet" OR "freshly made dog" OR "cooked pet food" OR "cooked diet" OR butternut OR butternutbox OR "butter nut" OR "different dog diet" OR differentdog OR "different dog food" OR "different dog meal" OR "dif dog" OR "diffrent dog" OR "monty's larder" OR "montys larder" OR montyslarder OR montys OR "monty larder" OR "boost pet" OR "boost dog" OR "pets love fresh" OR petslovefresh OR "pets lovefresh" OR "petslove fresh" OR fidelis OR fedelis OR "years dog food" OR "years pet food") AND ("health issue" OR "health issues" OR "skin issue" OR "skin issues" OR coat OR "digestive issue" OR "digestive issues" OR "low energy" OR lethargic OR lethargy OR "weight gain" OR "weight gains" OR obese OR obeisty OR obesity OR allergy OR allergys OR allergies OR allergic OR alergy OR alergys OR alergies OR alergic OR alergy OR allegy OR intolerance OR intolerances OR intollerance OR intollerances OR intolerent OR intolerant OR itch OR itchy OR itches OR itching OR itcy OR itchey OR scratching OR scratch OR scratchs OR scratches OR scratched OR scracth OR scracths OR diarrhea OR diarhea OR diahrea OR diarrea OR diarreah OR diahorrea OR diarrhoea OR diarhoea OR diarrheas OR diarrhoeas OR vomit OR vomits OR vomiting OR vomited OR vommit OR vommiting OR vommitted OR "flaky skin" OR "flakey skin" OR dermatitis OR dermititis OR "dry skin" OR infection OR infections OR infectious OR "soft feces" OR "soft faeces" OR "nasty poo" OR smelly OR fart OR farts OR farting OR farted OR faart OR faarts OR faarting OR flatulence OR flatulent OR "anal gland" OR "anal glands" OR "anel gland" OR "anel glands" OR analgland OR scoot OR scoots OR scooting OR scooted OR "back end" OR back-end OR dehydration OR dehydrated OR dehydrate OR dehydrates OR dehydrating OR "poor hydration" OR dull OR bloat OR bloats OR bloated OR bloating OR "upset stomach" OR "upset stomachs" OR "sensitive stomach" OR "sensitive stomachs" OR "sensitive tummy" OR "sensitive tummies" OR "sensitive tum" OR "upset tummy" OR "upset tummies" OR "upset tum" OR "hypoallergenic" OR "fussy eater" OR "picky eater" OR sick OR sickness OR sickly OR tarter OR tartar OR "soft stool" OR "soft stools" OR "runny poo" OR "runny poop" OR "loose poo" OR "loose poop") AND NOT (survey OR participant OR participants OR recruitment OR transgender OR trans OR interview OR "google form") AND NOT RETWEET

(B)

(LANG en) AND (LOCATION GB) AND (dog OR dogs OR puppy OR puppies OR pup OR pooch OR pooches OR "fur baby" OR "fur babies" OR furbaby OR furbabies OR hound OR hounds OR "my boy" OR "my girl") AND ("dog food" OR dogfood OR "pet food" OR "dog meals" OR "dog meal" OR "pet meal" OR "pet meals" OR "senior food" OR "senior diet" OR "puppy food" OR "puppy diet") AND ("fresh food" OR "fresh dog food" OR "fresh pet food" OR "fresh diet" OR "minimally processed" OR "minimally-processed" OR "human-grade" OR "human grade" OR "human quality" OR "gently cooked" OR "frozen dog" OR " frozen pet" OR "cold pressed" OR "refrigerated dog food" OR "refrigerated pet food" OR "refrigerated diet" OR "natural diet" OR "natural dog food" OR "natural pet food" OR "freshly made pet" OR "freshly made dog" OR "cooked pet food" OR "cooked diet" OR butternut OR butternutbox OR "butter nut" OR "different dog diet" OR differentdog OR "different dog food" OR "different dog meal" OR "dif dog" OR "diffrent dog" OR "monty's larder" OR "montys larder" OR montyslarder OR montys OR "monty larder" OR "boost pet" OR "boost dog" OR "pets love fresh" OR petslovefresh OR "pets lovefresh" OR "petslove fresh" OR fidelis OR fedelis OR "years dog food" OR "years pet food") AND (improve OR improves OR improved OR improving OR improvement OR improvements OR improved OR benefit OR benefits OR benefited OR benefitted OR benefiting OR benefitting OR better OR digest OR digests OR digesting OR digested OR digestion OR "gut health" OR firm OR firmer OR firming OR "more normal" OR "easier to pick up" OR healthier OR "less fart" OR "less farts" OR "less farting" OR "less flatulence" OR shinier OR shiny OR shininess OR gloss OR glossy OR glossiness OR soft OR softer OR softest OR softness OR "clear skin" OR itch OR itching OR itchy OR itches OR "less scratching" OR "reduced allergy" OR "reduced allergies" OR hypoallergen OR hypoallergenic OR hypoallergy OR hypoalergic OR energy OR energies OR energetic OR energetically OR vitality OR vital OR weight OR weight OR slim OR slimmer OR slimmest OR slimming OR "more active" OR playful OR playfully OR playfulness OR playfull OR activity OR activities OR calm OR calming OR calmer OR calmest OR calmness OR live OR lives OR lived OR living OR livelier OR liveliest OR happier OR happiness OR thriving OR thrive OR thrives OR thrived OR hydrate OR hydrates OR hydrated OR hydrating OR hydration OR appetite OR appetites OR appetitive OR appetit OR appetizing OR appetising OR apeti OR "less fussy" OR "less picky" OR breath OR breaths OR breathing OR breathe OR tummy OR tummies OR stomach OR sensitive OR poo OR poos OR stool OR stools OR waste OR coat OR "licking the bowl" OR "licks the bowl" OR "bowl clean") AND NOT (survey OR participant OR participants OR recruitment OR transgender OR trans OR interview OR "google form") AND NOT RETWEET

Figure S3. Temporal distribution of raw KC and FD social media posts. Lines show the number of posts per month in each raw dataset before relevance filtering and downstream analysis. N = 21,896 KC and 2,336 FD. KC, kibble/canned diets; FD, fresh diets including human-grade, gently/mildly cooked or home-cooked diets.

Figure S4. Data cleaning workflow to prepare raw data for relevance zero-shot classification run.

Figure S5. Character-length distributions of KC and FD posts in pilot (A) KC (n=9,924) and (B) FD (n=2,109) datasets. Based on visual inspection and manual spot checks, final inclusion ranges were set at 100–1300 characters for KC and 50–1700 characters for FD. These thresholds were subsequently applied to the full dataset. KC, kibble/canned diets; FD, fresh diets including human-grade, gently/mildly cooked or home-cooked diets.

Figure S6. Zero-shot classification fixed description prompt for LLM for KC and FD dataset posts classification as either relevant or irrelevant. KC, kibble/canned diets; FD, fresh diets including human-grade, gently/mildly cooked or home-cooked diets.

A post should be considered Relevant (R) when it clearly relates to both **dogs** and dog food or diets, and also contains some kind of **explicit health, outcome, or feeding-related observation** about the dog. The type of diet does not matter—it can include fresh, minimally processed, human-grade, gently cooked, organic, natural, raw, home-made, kibble, canned, or vet-prescribed diets, as well as specific brands such as Butternut Box, Different Dog, Tuggs, Royal Canin, Hills, Purina, Freshpet, or any other. Indirect or narrative references to feeding also count *only when they are tied to an observed effect or outcome*. For example, a post that says “we cook for him at home and his stools improved,” “she finally eats now,” or “we had to switch to another brand and his skin got worse” should be considered Relevant.

To qualify as Relevant, the post must also include at least one of the following elements: it mentions a dog-specific health benefit, concern, or condition (such as diarrhoea, constipation, firmer stools, itchy skin, allergies, changes in appetite, coat quality, dental health, weight, or longevity) **in relation to what the dog is fed**; it discusses switching diets and the reasons for switching **with reference to the dog’s health, appetite, or behavior**; it describes outcomes observed by the owner after feeding a diet (positive, negative, or neutral); or it refers to fussy/picky eating behavior (refusing food, losing appetite, finally eating again) **as a direct effect of the diet or feeding practice**. Both explicit outcomes (“her coat is shinier,” “his digestion improved,” “he has more energy”) and implicit outcomes (“he’s so much better now since switching food,” “she finally started eating with the new diet”) should be included as Relevant. Posts that mention multiple species can also be counted as Relevant **only if the dog-related portion clearly meets these criteria. Posts that are exclusively about other species (e.g., cats, including specific breeds such as Bengals, or other pets) should be considered Irrelevant.**

On the other hand, a post should be classified as Irrelevant (IR) when it is not about dogs at all, or when it mentions dogs but contains no clear connection to the food/diet fed **and** an observed health/outcome effect. The following types of content should also be considered Irrelevant:

- Posts that only discuss the **price, cost, or availability** of food without mentioning health, wellbeing, switching, or outcomes.

- Posts that only mention a **dog food brand, ingredient, or feeding practice** but make no link to the health, outcomes, or observed effects in the dog.

- Posts that are just **general advice, opinions, or cultural commentary** about feeding, recipes, or nutrition (e.g., “rice and chicken is common when dogs are sick,” “kibble quality varies,” “dog food storage tips”) without an explicit tie to a specific dog’s feeding and health outcome.

- **Species mismatches** (e.g., cats, Bengals, or other non-dog pets) unless the dog-specific portion includes clear outcome-based observations.

- **Product reviews, promotional material, or marketing content** where the main purpose is to promote/describe food and not to report an owner-observed outcome in their dog.

- Posts that are about **politics, recruitment, surveys, bots, fiction/role-play, or NSFW/sexual content.**

Figure S7. Large language model prompt for NMF topic interpretation. Prompt used for large language model-assisted interpretation of non-negative matrix factorization (NMF) topics. The model was provided with top weighted NMF terms and representative posts for each topic and asked to generate a topic label, summary, sentiment classification, exemplar quote, and ranked representative examples.

Figure S8. Exploratory post sentiment classification prompt. Prompt used for large language model-assisted exploratory sentiment classification of KC and FD posts as positive, negative, or neutral in relation to dog diet and owner-reported health-related signs. KC, kibble/canned diets; FD, fresh diets including human-grade, gently/mildly cooked or home-cooked diets.

"You are a qualitative researcher analyzing dog owners' perceptions on how diet impacts their dogs' health.\n"

"For each topic, provide:\n"

"1. Label: A descriptive health-theme label (can be longer than 5 words when needed).\n"

"2. Summary: (2-3 sentences on owner perceptions).\n"

"3. Topic_Sentiment: [Positive, Negative, or Neutral].\n"

"4. Exemplar_Quote: The best sentence from the samples that captures the theme.\n"

f"5. top_examples: list of the {top_n_examples} best full posts from the provided samples, ranked best-first.\n\n"

"Return as a JSON object with a 'topics' list. Do not hallucinate. "

"Only use text from provided samples for top_examples. "

Figure S9. NMF model-selection metrics across candidate topic numbers (4-15) for the KC and FD datasets. Panels show coherence (Cv, NPMI, and UMass), mean stability across repeated runs, topic redundancy, topic uniqueness, and reconstruction error across tested values of k. Vertical reference lines indicate the final selected models (KC: k = 7; FD: k = 6). KC, kibble/canned diets; FD, fresh diets including human-grade, gently/mildly cooked or home-cooked diets; Cv, topic coherence; NPMI, normalized pointwise mutual information.

Figure S10. Sentiment distribution across topic-modelled themes for (A) kibble/canned diets (KC) posts (n = 8,040) and (B) fresh diet (FD) posts (n = 924). Bars represent the proportion of posts classified by sentiment (e.g. positive, negative, neutral) within each topic. Sentiment classification was performed by prompting a large language model (GPT-4o mini, OpenAI). GI, gastrointestinal; FD, fresh diets including human-grade, gently/mildly cooked or home-cooked diets.

Figure S11. Owner-reported health-related signs (ORHS) quantification dictionary and coding rules. Summary of the rule-based ORHS quantification framework used to identify owner-reported observable signs language in social media posts.

**Coding rules**

- Text was quantified at post level using full_text (title and content concatenated).
- Categories were non-mutually exclusive and coded as binary presence/absence per post.
- Repeated matches within the same domain and polarity were counted once per post; unique evidence terms were retained.
- Negation and improvement heuristics were applied so some concern phrases could contribute to benefit coding only when context indicated improvement.
- Sentence boundaries from punctuation were used where available to limit cross-sentence linking of improvement language.
- Product-name and non-health exclusions were applied to reduce false positives from ambiguous terms.
- Key custom exclusions included ‘Bernie's Perfect Poop’ for GI health benefit perfect poo* and object-moving uses of scoot* (e.g. scoot it, scoot bowl) for GI concern.
- Broad concern phrases were prevented from flipping to benefit in selected categories, particularly GI health, appetite/fussy eating, illness, and allergy/intolerance.

**Gastrointestinal health**

**Concern terms**

diarrhoea, diarrhea, explosive diarrhoea, explosive diarrhea, loose stool, loose stools, soft stool, soft stools, soft poo*, soft stool*, runny poo*, runny stool*, mushy stool*, soft feces, soft faeces, loose poo*, nasty poo*, poo* is too soft, vomit*, throwing up, threw up, vomiting everything, being sick, make him sick, make her sick, made him sick, made her sick, sick after eating, feels sick, feeling sick, nausea, nauseous, regurgitat*, dry heave, heaving, gagging, acid reflux, heartburn, burp, belch, burping, belching, constipat*, gassy, flatulence, windy, fart*, farting a lot, lots of farts, bad gas, a lot of gas, lots of gas, has gas, gives him gas, gives her gas, passing gas, bloat*, upset stomach, upset tummy, tummy issues, tummy troubles, tummy problems, gi upset, gi issues, gi problems, gi symptoms, gastro issues, gastro upset, gastro reaction, gastrointestinal tract, gastrointestinal track, gastrointestinal upset, gastrointestinal disease, gastrointestinal intolerance, gastrointestinal issues, gastrointestinal problems, gastritis, wonky tummy, off tummy, upset stomachs, upset tummies, upset tum, tummy isnt what it used to be, runny bum, sensitive digestion, sensitive guts, sensitive stomach, sensitive tummy, sensitive tum*, sensitive stomachs, anxious stomach, stomach ache, tummy ache, rumbling tummy, tummy rumbling, stomach acting up, stomach is acting up, digestive issues, anal gland, anal glands, anal sac, anal sacs, impacted glands, gland impaction, infected glands, gland infection, ruptured gland, leaking glands, fishy smell from bum, fishy smell from butt, scoot*, butt scoot, scoots his bum, scoots her bum, dragging bum, dragging butt, licking bum, licking butt, chronic gas, gas is terrible, gas is awful, gas is horrible, so much gas, terrible gas, gut bacteria issues, gut bacteria problems, bad gut bacteria, unhealthy gut bacteria, gut microbiome issues, gut microbiome disrupted, disrupted gut flora, colitis, ibd, ibs, inflammatory bowel disease, pancreatitis, bloody stool*, blood in stool, bloody poo*, blood filled poo*, haematochezia, hematochezia, melena, melaena, mucus in stool, mucous in stool, pooping mucus, yellow stool, black stool, pale stool, greasy stool, messy stool

**Benefit terms**

improved digestion, better digestion, digestion improved, digestion better, bowel movements improved, poos improved, stools improved, better stool, healthier stool, poop improved, poops improved, poop is better, more normal poo, normal stools, perfect poo*, regular poo*, firmer stool*, stools are firm, formed stool, formed stools, well formed stool*, solid stool*, easier to pick up, easy to pick up, less fart*, farts less, less gassy, less flatulence, settled stomach, settled tummy, calmer tummy, healthier tummy, healthy poop, healthy poops, never upset his tum*, never upset her tum*, never upset my girls tum*, fine for his digestion, fine for her digestion, easily digested, more easily digested, poo* look healthy, coat and poop* look, stool returned to normal, system back on track, hasnt had any stomach problems, no regurgitation, stopped vomiting, no more vomiting, not throwing it up anymore, not vomiting, no longer vomiting, hasnt vomited, havent vomited, never vomits, not been sick since, not thrown up, hasnt thrown up, havent thrown up, never thrown up, glands clear, anal glands fine, no more scooting, stopped scooting, no fishy smell from bum, no fishy smell from butt, smell gone, poop less, poops less, smaller poops

**Skin/coat**

**Concern terms**

itch*, scratch*, scratches a lot, rash, rashes, hotspot, hotspots, hot spot, hot spots, redness, red skin, inflamed skin, sores, sore skin, dermatitis, atopy, atopic dermatitis, dandruff, flak* skin, dry skin, scabs, crusty skin, oozing skin, greasy coat, alopecia, hair loss, patchy fur, pruritus, hives, welts, yeast*, yeasty smell, malassezia, red paws, sore paws, paw licking, paw chewing, chewing his paws, chewing her paws, lick granuloma, skin infection, skin infections, skin issues, skin problems, skin issue, coat isnt shiny, turned red, obsessive licking, skin allerg*

**Benefit terms**

shiny coat, shinier coat, shiny fur, glossy coat, glossier coat, soft coat, softer coat, soft fur, softer fur, clear skin, skin cleared, skin cleared up, skin improved, skin better, less scratching, scratching less, reduced itching, stopped scratching, itch free, itch-free, less itchy, reduced allergy, reduced allergies, healthy skin, skin looks healthy, healthy coat, healthier coat, coat looks healthy, coat looks better, reduced shedding, less shedding, less dandruff, coat improved, coat condition improved, fur softer, full coat, coat healthy, improved itchy skin, fewer skin issues, coat is amazing, no more hotspots, no more hot spots

**Ear/eye**

**Concern terms**

otitis, otitis externa, ear infection, ear infections, eye infection, eye infections, infected ears, infected eye, infected eyes, ear flare, ear flares, ear flare up, ear flare-up, yeasty ears, yeast in ears, smelly ears, stinky ears, ear mites, head shaking, shaking his head, shaking her head, ear discharge, gunky ears, goopy ears, ear gunk, brown ear discharge, runny eyes, eye discharge, gunky eyes, goopy eyes, conjunctivitis, tear stains, tear staining, eye stains, eye staining, teary eyes, watery eyes, tearing, epiphora, dry eye, red eyes, sore eyes, itchy ears, ear problems, ear condition

**Benefit terms**

clear eyes, eyes cleared up, clean ears, ears cleaned up, less head shaking, no more head shaking, healthier ears, healthier eyes, no tear stains, tear stains gone, less discharge, discharge reduced, fewer ear infections, fewer eye infections, ear infection* disappeared, ear problem* disappeared, ear issue* disappeared, no more ear infection*, no more ear problem*

**Allergy/intolerance**

**Concern terms**

allerg*, intolerance, intolerances, food intolerance*, food sensitivit*, food sensitive, intolerant, reaction*, adverse reaction, flare-up, flare up, flare ups, histamine, intolerant to, cannot tolerate, trigger food, triggered by food, allergic reaction, allergen, allergens, less tolerant, sensitive to it, storage mite, storage mites, grain allergy, chicken allergy

**Benefit terms**

reduced allergy, reduced allergies, less allergic, fewer reactions, reactions stopped, improved tolerance, better tolerance, better tolerating, now tolerating, tolerating well, tolerates it well, tolerating it well, tolerating the food, less sensitive, symptom free, symptom-free, flare settled, flares settled, allergies are nonexistant, allergies are nonexistent, no food related allergies, never developed food related allergies

**Weight/body condition**

**Concern terms**

overweight, obese, obesity, chonky, chonk, chunky, pudgy, too heavy, too much weight, weight gain, gained weight, piling on weight, gaining weight, put on weight, getting fatter, fatter, over weight, bit of chub, little potato, poor condition, poor body condition, bcs high, body condition score high, love handles, underweight, skinny, ribs showing, too thin, too skinny, lost too much weight, significant weight loss, caloric recom*, over fed, underfed, under fed, cant gain weight, can't gain weight, trouble gaining weight, trouble keeping weight, struggle* to gain weight, struggle* to put weight, failing to gain weight, not gaining weight, isnt gaining weight, wont put weight on, cant keep weight on, having trouble keeping her weight up, having trouble keeping his weight up

**Benefit terms**

slimming, leaner, keeps him trim, keeps her trim, stayed trim, healthy weight, at a healthy weight, ideal weight, at ideal weight, maintain weight, maintained weight, weight stable, weight is stable, weight stabilized, weight stabilised, normal weight, weight he should be, weight she should be, helping weight management, most fit, put weight on, kept weight on, keep weight on, weight up, better condition, good condition, more muscle, muscle mass improved, bcs improved, body condition score improved, ribs not visible, filled out

**Energy/behavior**

**Concern terms**

lethargic, so lethargic, tired all the time, always tired, very tired, low energy, no energy, less energy, lethargy, lethary, lazy, inactive, anxious, very anxious, aggression, aggressive, hyper, hyperactive, stressed, stressy, restless, can't settle, cant settle, reactive, nervy, on edge, overaroused, panting at rest, panting a lot, whining, whines a lot

**Benefit terms**

energetic, more energetic, playful, more playful, more active, very active, activity level up, more energy, loads of energy, energy levels improved, energy levels up, happier, seems happier, looks happier, he is thriving, hes thriving, she is thriving, shes thriving, they are thriving, theyre thriving, im thriving, thrive on it, calm, calmer, super calm, more settled, more focused, less anxious, less nervous, more confident, better mood, brighter, more bright, perked up, vitality, livelier, massive resurgence of energy, resurgence of energy, zoomies more

**Dental/oral**

**Concern terms**

bad breath, stinky breath, smelly breath, halitosis, tartar, tartar build up, tartar buildup, tarter, plaque, plaque build up, plaque buildup, gum disease, gingivitis, sore gums, teeth issues, bad teeth, dental disease, dental surgery, tooth decay, rotten teeth, rotting teeth, broken tooth, broken teeth, teeth removed, tooth removed, oronasal fistula, brown teeth, yellow teeth, breath smells bad

**Benefit terms**

clean teeth, really clean teeth, whiter teeth, white teeth, fresher breath, better breath, less tartar, tartar reduced, less plaque, plaque reduced, healthy gums, gums look healthy, never had dental problems, never had any dental problems, no dental problems, hasnt had dental problems, hasnt had any dental problems

**Appetite/fussy eating**

**Concern terms**

fussy eater, picky eater, picky eating, pickiness, fussy with food, picky with food, not eating, wont eat, stopped eating, won't touch food, wont touch food, barely eats, refus* to eat, refus* eat, refus* food, went off food, off his food, off her food, poor appetite, loss of appetite, decreased appetite, appetite decreased, appetite dropped, worsening appetite, lost his appetite, lost her appetite, reduced appetite, no appetite, finicky, not food motivated, food aversion, food refusal, picks at food, picking at food, just picks, nibbles, only nibbles, turns nose up, sniffs and walks away, grazing, food strike, on a hunger strike, skipping meals, misses meals, barely touches his food, barely touches her food, doesnt like it, not food motivated, not super food motivated, not supper food motivated, goes off it, wouldnt even sniff, turning her nose, turning his nose, eating issues, averted his food, averted her food, fussi*

**Benefit terms**

better appetite, improved appetite, good appetite, less fussy, less picky, licking the bowl, licks the bowl, bowl clean, clean bowl, cleans the bowl, finishes bowl, finishes meals, finishes his meals, finishes her meals, eats more, eating more, finally eating, eating on his own, eating on her own, eats everything, eat anything, eating better, eating well, eating again, eats eagerly, gobbles, gobbling his food, gobbling her food, gobble up their food, gobbled it up, eagerly gobble*, wolfing food, wolfs his food, wolfs her food

**General health/illness**

**Concern terms**

poorly, often sick, sick a lot, infection, infected, bladder infection, uti, fever, high temperature, collaps*, seizure*, fits, giardia, worm*, parasit*, tumour*, tumor*, cancer, heart disease, dcm, diabetes, developing diabetes, dementia, lymphoma, enucleation, mucus, snotty, on antibiotics, antibiotic course, hospitalised, hospitalized, in hospital

**Benefit terms**

recovery, recovered, recovering, fully recovered, in remission, infection cleared, infection gone, illness resolved, illness cleared, back to full health, back to good health, no longer sick, not sick anymore, symptoms improved, symptoms resolved

improved, poos improved, stools improved, better stool, healthier stool, poop improved, poops improved, poop is better, more normal poo, normal stools, perfect poo*, regular poo*, firmer stool*, stools are firm, formed stool, formed stools, well formed stool*, solid stool*, easier to pick up, easy to pick up, less fart*, farts less, less gassy, less flatulence, settled stomach, settled tummy, calmer tummy, healthier tummy, healthy poop, healthy poops, never upset his tum*, never upset her tum*, never upset my girls tum*, fine for his digestion, fine for her digestion, easily digested, more easily digested, poo* look healthy, coat and poop* look, stool returned to normal, system back on track, hasnt had any stomach problems, no regurgitation, stopped vomiting, no more vomiting, not throwing it up anymore, not vomiting, no longer vomiting, hasnt vomited, havent vomited, never vomits, not been sick since, not thrown up, hasnt thrown up, havent thrown up, never thrown up, glands clear, anal glands fine, no more scooting, stopped scooting, no fishy smell from bum, no fishy smell from butt, smell gone, poop less, poops less, smaller poops

**Skin/coat**

**Concern terms**

itch*, scratch*, scratches a lot, rash, rashes, hotspot, hotspots, hot spot, hot spots, redness, red skin, inflamed skin, sores, sore skin, dermatitis, atopy, atopic dermatitis, dandruff, flak* skin, dry skin, scabs, crusty skin, oozing skin, greasy coat, alopecia, hair loss, patchy fur, pruritus, hives, welts, yeast*, yeasty smell, malassezia, red paws, sore paws, paw licking, paw chewing, chewing his paws, chewing her paws, lick granuloma, skin infection, skin infections, skin issues, skin problems, skin issue, coat isnt shiny, turned red, obsessive licking, skin allerg*

**Benefit terms**

shiny coat, shinier coat, shiny fur, glossy coat, glossier coat, soft coat, softer coat, soft fur, softer fur, clear skin, skin cleared, skin cleared up, skin improved, skin better, less scratching, scratching less, reduced itching, stopped scratching, itch free, itch-free, less itchy, reduced allergy, reduced allergies, healthy skin, skin looks healthy, healthy coat, healthier coat, coat looks healthy, coat looks better, reduced shedding, less shedding, less dandruff, coat improved, coat condition improved, fur softer, full coat, coat healthy, improved itchy skin, fewer skin issues, coat is amazing, no more hotspots, no more hot spots

**Ear/eye**

**Concern terms**

otitis, otitis externa, ear infection, ear infections, eye infection, eye infections, infected ears, infected eye, infected eyes, ear flare, ear flares, ear flare up, ear flare-up, yeasty ears, yeast in ears, smelly ears, stinky ears, ear mites, head shaking, shaking his head, shaking her head, ear discharge, gunky ears, goopy ears, ear gunk, brown ear discharge, runny eyes, eye discharge, gunky eyes, goopy eyes, conjunctivitis, tear stains, tear staining, eye stains, eye staining, teary eyes, watery eyes, tearing, epiphora, dry eye, red eyes, sore eyes, itchy ears, ear problems, ear condition

**Benefit terms**

clear eyes, eyes cleared up, clean ears, ears cleaned up, less head shaking, no more head shaking, healthier ears, healthier eyes, no tear stains, tear stains gone, less discharge, discharge reduced, fewer ear infections, fewer eye infections, ear infection* disappeared, ear problem* disappeared, ear issue* disappeared, no more ear infection*, no more ear problem*

**Allergy/intolerance**

**Concern terms**

allerg*, intolerance, intolerances, food intolerance*, food sensitivit*, food sensitive, intolerant, reaction*, adverse reaction, flare-up, flare up, flare ups, histamine, intolerant to, cannot tolerate, trigger food, triggered by food, allergic reaction, allergen, allergens, less tolerant, sensitive to it, storage mite, storage mites, grain allergy, chicken allergy

**Benefit terms**

reduced allergy, reduced allergies, less allergic, fewer reactions, reactions stopped, improved tolerance, better tolerance, better tolerating, now tolerating, tolerating well, tolerates it well, tolerating it well, tolerating the food, less sensitive, symptom free, symptom-free, flare settled, flares settled, allergies are nonexistant, allergies are nonexistent, no food related allergies, never developed food related allergies

**Weight/body condition**

**Concern terms**

overweight, obese, obesity, chonky, chonk, chunky, pudgy, too heavy, too much weight, weight gain, gained weight, piling on weight, gaining weight, put on weight, getting fatter, fatter, over weight, bit of chub, little potato, poor condition, poor body condition, bcs high, body condition score high, love handles, underweight, skinny, ribs showing, too thin, too skinny, lost too much weight, significant weight loss, caloric recom*, over fed, underfed, under fed, cant gain weight, can't gain weight, trouble gaining weight, trouble keeping weight, struggle* to gain weight, struggle* to put weight, failing to gain weight, not gaining weight, isnt gaining weight, wont put weight on, cant keep weight on, having trouble keeping her weight up, having trouble keeping his weight up

**Benefit terms**

slimming, leaner, keeps him trim, keeps her trim, stayed trim, healthy weight, at a healthy weight, ideal weight, at ideal weight, maintain weight, maintained weight, weight stable, weight is stable, weight stabilized, weight stabilised, normal weight, weight he should be, weight she should be, helping weight management, most fit, put weight on, kept weight on, keep weight on, weight up, better condition, good condition, more muscle, muscle mass improved, bcs improved, body condition score improved, ribs not visible, filled out

**Energy/behavior**

**Concern terms**

lethargic, so lethargic, tired all the time, always tired, very tired, low energy, no energy, less energy, lethargy, lethary, lazy, inactive, anxious, very anxious, aggression, aggressive, hyper, hyperactive, stressed, stressy, restless, can't settle, cant settle, reactive, nervy, on edge, overaroused, panting at rest, panting a lot, whining, whines a lot

**Benefit terms**

energetic, more energetic, playful, more playful, more active, very active, activity level up, more energy, loads of energy, energy levels improved, energy levels up, happier, seems happier, looks happier, he is thriving, hes thriving, she is thriving, shes thriving, they are thriving, theyre thriving, im thriving, thrive on it, calm, calmer, super calm, more settled, more focused, less anxious, less nervous, more confident, better mood, brighter, more bright, perked up, vitality, livelier, massive resurgence of energy, resurgence of energy, zoomies more

**Dental/oral**

**Concern terms**

bad breath, stinky breath, smelly breath, halitosis, tartar, tartar build up, tartar buildup, tarter, plaque, plaque build up, plaque buildup, gum disease, gingivitis, sore gums, teeth issues, bad teeth, dental disease, dental surgery, tooth decay, rotten teeth, rotting teeth, broken tooth, broken teeth, teeth removed, tooth removed, oronasal fistula, brown teeth, yellow teeth, breath smells bad

**Benefit terms**

clean teeth, really clean teeth, whiter teeth, white teeth, fresher breath, better breath, less tartar, tartar reduced, less plaque, plaque reduced, healthy gums, gums look healthy, never had dental problems, never had any dental problems, no dental problems, hasnt had dental problems, hasnt had any dental problems

**Appetite/fussy eating**

**Concern terms**

fussy eater, picky eater, picky eating, pickiness, fussy with food, picky with food, not eating, wont eat, stopped eating, won't touch food, wont touch food, barely eats, refus* to eat, refus* eat, refus* food, went off food, off his food, off her food, poor appetite, loss of appetite, decreased appetite, appetite decreased, appetite dropped, worsening appetite, lost his appetite, lost her appetite, reduced appetite, no appetite, finicky, not food motivated, food aversion, food refusal, picks at food, picking at food, just picks, nibbles, only nibbles, turns nose up, sniffs and walks away, grazing, food strike, on a hunger strike, skipping meals, misses meals, barely touches his food, barely touches her food, doesnt like it, not food motivated, not super food motivated, not supper food motivated, goes off it, wouldnt even sniff, turning her nose, turning his nose, eating issues, averted his food, averted her food, fussi*

**Benefit terms**

better appetite, improved appetite, good appetite, less fussy, less picky, licking the bowl, licks the bowl, bowl clean, clean bowl, cleans the bowl, finishes bowl, finishes meals, finishes his meals, finishes her meals, eats more, eating more, finally eating, eating on his own, eating on her own, eats everything, eat anything, eating better, eating well, eating again, eats eagerly, gobbles, gobbling his food, gobbling her food, gobble up their food, gobbled it up, eagerly gobble*, wolfing food, wolfs his food, wolfs her food

**General health/illness**

**Concern terms**

poorly, often sick, sick a lot, infection, infected, bladder infection, uti, fever, high temperature, collaps*, seizure*, fits, giardia, worm*, parasit*, tumour*, tumor*, cancer, heart disease, dcm, diabetes, developing diabetes, dementia, lymphoma, enucleation, mucus, snotty, on antibiotics, antibiotic course, hospitalised, hospitalized, in hospital

**Benefit terms**

recovery, recovered, recovering, fully recovered, in remission, infection cleared, infection gone, illness resolved, illness cleared, back to full health, back to good health, no longer sick, not sick anymore, symptoms improved, symptoms resolved

**Benefit terms**

reduced allergy, reduced allergies, less allergic, fewer reactions, reactions stopped, improved tolerance, better tolerance, better tolerating, now tolerating, tolerating well, tolerates it well, tolerating it well, tolerating the food, less sensitive, symptom free, symptom-free, flare settled, flares settled, allergies are nonexistant, allergies are nonexistent, no food related allergies, never developed food related allergies

**Weight/body condition**

**Concern terms**

overweight, obese, obesity, chonky, chonk, chunky, pudgy, too heavy, too much weight, weight gain, gained weight, piling on weight, gaining weight, put on weight, getting fatter, fatter, over weight, bit of chub, little potato, poor condition, poor body condition, bcs high, body condition score high, love handles, underweight, skinny, ribs showing, too thin, too skinny, lost too much weight, significant weight loss, caloric recom*, over fed, underfed, under fed, cant gain weight, can't gain weight, trouble gaining weight, trouble keeping weight, struggle* to gain weight, struggle* to put weight, failing to gain weight, not gaining weight, isnt gaining weight, wont put weight on, cant keep weight on, having trouble keeping her weight up, having trouble keeping his weight up

**Benefit terms**

slimming, leaner, keeps him trim, keeps her trim, stayed trim, healthy weight, at a healthy weight, ideal weight, at ideal weight, maintain weight, maintained weight, weight stable, weight is stable, weight stabilized, weight stabilised, normal weight, weight he should be, weight she should be, helping weight management, most fit, put weight on, kept weight on, keep weight on, weight up, better condition, good condition, more muscle, muscle mass improved, bcs improved, body condition score improved, ribs not visible, filled out

**Energy/behavior**

**Concern terms**

lethargic, so lethargic, tired all the time, always tired, very tired, low energy, no energy, less energy, lethargy, lethary, lazy, inactive, anxious, very anxious, aggression, aggressive, hyper, hyperactive, stressed, stressy, restless, can't settle, cant settle, reactive, nervy, on edge, overaroused, panting at rest, panting a lot, whining, whines a lot

**Benefit terms**

energetic, more energetic, playful, more playful, more active, very active, activity level up, more energy, loads of energy, energy levels improved, energy levels up, happier, seems happier, looks happier, he is thriving, hes thriving, she is thriving, shes thriving, they are thriving, theyre thriving, im thriving, thrive on it, calm, calmer, super calm, more settled, more focused, less anxious, less nervous, more confident, better mood, brighter, more bright, perked up, vitality, livelier, massive resurgence of energy, resurgence of energy, zoomies more

**Dental/oral**

**Concern terms**

bad breath, stinky breath, smelly breath, halitosis, tartar, tartar build up, tartar buildup, tarter, plaque, plaque build up, plaque buildup, gum disease, gingivitis, sore gums, teeth issues, bad teeth, dental disease, dental surgery, tooth decay, rotten teeth, rotting teeth, broken tooth, broken teeth, teeth removed, tooth removed, oronasal fistula, brown teeth, yellow teeth, breath smells bad

**Benefit terms**

clean teeth, really clean teeth, whiter teeth, white teeth, fresher breath, better breath, less tartar, tartar reduced, less plaque, plaque reduced, healthy gums, gums look healthy, never had dental problems, never had any dental problems, no dental problems, hasnt had dental problems, hasnt had any dental problems

**Appetite/fussy eating**

**Concern terms**

fussy eater, picky eater, picky eating, pickiness, fussy with food, picky with food, not eating, wont eat, stopped eating, won't touch food, wont touch food, barely eats, refus* to eat, refus* eat, refus* food, went off food, off his food, off her food, poor appetite, loss of appetite, decreased appetite, appetite decreased, appetite dropped, worsening appetite, lost his appetite, lost her appetite, reduced appetite, no appetite, finicky, not food motivated, food aversion, food refusal, picks at food, picking at food, just picks, nibbles, only nibbles, turns nose up, sniffs and walks away, grazing, food strike, on a hunger strike, skipping meals, misses meals, barely touches his food, barely touches her food, doesnt like it, not food motivated, not super food motivated, not supper food motivated, goes off it, wouldnt even sniff, turning her nose, turning his nose, eating issues, averted his food, averted her food, fussi*

**Benefit terms**

better appetite, improved appetite, good appetite, less fussy, less picky, licking the bowl, licks the bowl, bowl clean, clean bowl, cleans the bowl, finishes bowl, finishes meals, finishes his meals, finishes her meals, eats more, eating more, finally eating, eating on his own, eating on her own, eats everything, eat anything, eating better, eating well, eating again, eats eagerly, gobbles, gobbling his food, gobbling her food, gobble up their food, gobbled it up, eagerly gobble*, wolfing food, wolfs his food, wolfs her food

**General health/illness**

**Concern terms**

poorly, often sick, sick a lot, infection, infected, bladder infection, uti, fever, high temperature, collaps*, seizure*, fits, giardia, worm*, parasit*, tumour*, tumor*, cancer, heart disease, dcm, diabetes, developing diabetes, dementia, lymphoma, enucleation, mucus, snotty, on antibiotics, antibiotic course, hospitalised, hospitalized, in hospital

**Benefit terms**

recovery, recovered, recovering, fully recovered, in remission, infection cleared, infection gone, illness resolved, illness cleared, back to full health, back to good health, no longer sick, not sick anymore, symptoms improved, symptoms resolved

**Appetite/fussy eating**

**Concern terms**

fussy eater, picky eater, picky eating, pickiness, fussy with food, picky with food, not eating, wont eat, stopped eating, won't touch food, wont touch food, barely eats, refus* to eat, refus* eat, refus* food, went off food, off his food, off her food, poor appetite, loss of appetite, decreased appetite, appetite decreased, appetite dropped, worsening appetite, lost his appetite, lost her appetite, reduced appetite, no appetite, finicky, not food motivated, food aversion, food refusal, picks at food, picking at food, just picks, nibbles, only nibbles, turns nose up, sniffs and walks away, grazing, food strike, on a hunger strike, skipping meals, misses meals, barely touches his food, barely touches her food, doesnt like it, not food motivated, not super food motivated, not supper food motivated, goes off it, wouldnt even sniff, turning her nose, turning his nose, eating issues, averted his food, averted her food, fussi*

**Benefit terms**

better appetite, improved appetite, good appetite, less fussy, less picky, licking the bowl, licks the bowl, bowl clean, clean bowl, cleans the bowl, finishes bowl, finishes meals, finishes his meals, finishes her meals, eats more, eating more, finally eating, eating on his own, eating on her own, eats everything, eat anything, eating better, eating well, eating again, eats eagerly, gobbles, gobbling his food, gobbling her food, gobble up their food, gobbled it up, eagerly gobble*, wolfing food, wolfs his food, wolfs her food

**General health/illness**

**Concern terms**

poorly, often sick, sick a lot, infection, infected, bladder infection, uti, fever, high temperature, collaps*, seizure*, fits, giardia, worm*, parasit*, tumour*, tumor*, cancer, heart disease, dcm, diabetes, developing diabetes, dementia, lymphoma, enucleation, mucus, snotty, on antibiotics, antibiotic course, hospitalised, hospitalized, in hospital

**Benefit terms**

recovery, recovered, recovering, fully recovered, in remission, infection cleared, infection gone, illness resolved, illness cleared, back to full health, back to good health, no longer sick, not sick anymore, symptoms improved, symptoms resolved

Table S1. Metadata characteristics of the mined publicly available online social media posts. KC, kibble/canned diets; FD, fresh diets including human-grade, gently/mildly cooked or home-cooked diets.

| Variable | KC posts (n = 21,896) | FD posts (n = 2,336) |
| --- | --- | --- |
| Source |  |  |
| Reddit, n (%) | 21,130 (96.5) | 2,279 (97.6) |
| Other platforms, n (%) | 766 (3.5) | 57 (2.4) |
| Country (user-reported) |  |  |
| Present, n (%) | 628 (2.9) | 46 (2.0) |
| Missing, n (%) | 21,268 (97.1) | 2,290 (98.0) |
| Gender (user-reported) |  |  |
| Female, n (%) | 446 (2.0) | 28 (1.2) |
| Male, n (%) | 453 (2.1) | 39 (1.7) |
| Other/unknown, n (%) | 20,746 (94.7) | 2,257 (96.6) |
| Missing, n (%) | 251 (1.1) | 12 (0.5) |

Table S2: Inter-coder reliability statistics across kibble/caned diet (KC) posts and fresh diet (FD) posts. FD included human-grade, gently/mildly cooked or home-cooked diets.

| Dataset (posts) | N = | Agreement (%) | Cohen’s κ  (95% CI) | Gwet’s AC1  (95% CI) | PABAK  (95% CI) | Interpretation^a^ |
| --- | --- | --- | --- | --- | --- | --- |
| KC | 200 | 88.5 | 0.72 (0.61–0.82) | 0.81 (0.72–0.88) | 0.77 (0.68–0.85) | Substantial |
| FD | 141 | 90.1 | 0.80 (0.69–0.90) | 0.80 (0.70–0.90) | 0.80 (0.70–0.90) | Substantial |

^a^Interpretation based on Cohen’s κ = 0.61–0.80 = substantial agreement; κ = 0.81–1.00 = almost perfect agreement.

Table S3: Human vs large language model relevance classification performance statistics. A manually coded dataset by 2 reviewers was used as the ground truth for evaluating the performance of the LLM in relevance classification. KC, kibble/canned diets; FD, fresh diets including human-grade, gently/mildly cooked or home-cooked diets.

| Validation Type | Dataset / Set | Total N | Accuracy | Precision | Recall | F1 score |
| --- | --- | --- | --- | --- | --- | --- |
| Ground-truth validation (2 reviewers) | KC | 200 | 0.88 | 0.89 | 0.95 | 0.92 |
|  | FD | 141 | 0.90 | 0.93 | 0.90 | 0.91 |
| Single-reviewer validation (1 reviewer)  Random, time-stratified manual validation checks | KC – Set 1 | 145 | 0.95 | 0.96 | 0.98 | 0.97 |
|  | KC – Set 2 | 149 | 0.92 | 0.92 | 0.98 | 0.95 |
|  | KC – Set 3 | 150 | 0.93 | 0.95 | 0.96 | 0.95 |
|  | FD – Set 1 | 100 | 0.90 | 0.86 | 0.98 | 0.92 |
|  | FD – Set 2 | 50 | 0.86 | 0.80 | 0.91 | 0.85 |

Table S4: Validation metrics for the exploratory text-based health effect dictionary classifier. A manually annotated dataset (n = 100) was used as the reference standard to evaluate classifier performance at the category and polarity (concern or benefit) level. True positives (TP), false positives (FP), and false negatives (FN) were calculated for each category–polarity pair. Precision (positive predictive value, PPV) was defined as TP / (TP + FP), and recall (sensitivity) as TP / (TP + FN). The F1 score was calculated as the harmonic mean of precision and recall. The macro-averaged F1 score across 17 category–polarity pairs (excluding Energy/Behavior-Concern) was 0.950. This category was excluded from the macro-average because no reference standard positive instances were identified. All 95% confidence intervals (CI) are Wilson score intervals.

| Health effect | Polarity | TP | FP | FN | Precision [95% CI] | Recall [95% CI] | F1 |
| --- | --- | --- | --- | --- | --- | --- | --- |
| GI health | Concern | 32 | 1 | 2 | 0.970 [0.847, 0.995] | 0.941 [0.809, 0.984] | 0.955 |
| GI health | Benefit | 15 | 1 | 0 | 0.938 [0.717, 0.989] | 1.000 [0.796, 1.000] | 0.968 |
| Skin and coat | Concern | 12 | 0 | 0 | 1.000 [0.757, 1.000] | 1.000 [0.757, 1.000] | 1.000 |
| Skin and coat | Benefit | 3 | 1 | 1 | 0.750 [0.301, 0.954] | 0.750 [0.301, 0.954] | 0.750 |
| Ear and eye | Concern | 2 | 0 | 0 | 1.000 [0.342, 1.000] | 1.000 [0.342, 1.000] | 1.000 |
| Ear and eye | Benefit | 1 | 0 | 0 | 1.000 [0.207, 1.000] | 1.000 [0.207, 1.000] | 1.000 |
| Allergy and intolerance | Concern | 14 | 2 | 0 | 0.875 [0.640, 0.965] | 1.000 [0.785, 1.000] | 0.933 |
| Allergy and intolerance | Benefit | 3 | 1 | 1 | 0.750 [0.301, 0.954] | 0.750 [0.301, 0.954] | 0.750 |
| Weight and body condition | Concern | 10 | 0 | 0 | 1.000 [0.722, 1.000] | 1.000 [0.722, 1.000] | 1.000 |
| Weight and body condition | Benefit | 4 | 1 | 1 | 0.800 [0.376, 0.964] | 0.800 [0.376, 0.964] | 0.800 |
| Energy and behavior | Concern | 0 | 0 | 0 | - | - | - |
| Energy and behavior | Benefit | 4 | 0 | 0 | 1.000 [0.510, 1.000] | 1.000 [0.510, 1.000] | 1.000 |
| Dental and oral | Concern | 2 | 0 | 0 | 1.000 [0.342, 1.000] | 1.000 [0.342, 1.000] | 1.000 |
| Dental and oral | Benefit | 1 | 0 | 0 | 1.000 [0.207, 1.000] | 1.000 [0.207, 1.000] | 1.000 |
| Appetite and fussy | Concern | 20 | 0 | 0 | 1.000 [0.839, 1.000] | 1.000 [0.839, 1.000] | 1.000 |
| Appetite and fussy | Benefit | 2 | 0 | 0 | 1.000 [0.342, 1.000] | 1.000 [0.342, 1.000] | 1.000 |
| General health and illness | Concern | 17 | 0 | 0 | 1.000 [0.816, 1.000] | 1.000 [0.816, 1.000] | 1.000 |
| General health and illness | Benefit | 1 | 0 | 0 | 1.000 [0.207, 1.000] | 1.000 [0.207, 1.000] | 1.000 |
| Micro-average | All | 143 | 7 | 7 | 0.953 [0.907, 0.977] | 0.966 [0.923, 0.985] | 0.960 |

Table S5: Precision (positive predictive value, PPV) of the exploratory text-based health effect dictionary classifier using script-positive posts (n=547 from kibble/canned diet and fresh diet posts), assessed by a single annotator. Overall PPV was 0.91 (95% CI: 0.89–0.93), with a false positive rate of 0.09 (95% CI: 0.07–0.11). Category-specific PPV ranged from 0.83–1.00 (concern) and 0.46–1.00 (benefit). Micro-averaged PPV was calculated separately by polarity. TP, true positive; FP, false positive. All 95% confidence intervals (CI) are Wilson score intervals.  FD included human-grade, gently/mildly cooked or home-cooked diets.

| Health effect | Polarity | Posts reviewed | TP | FP | PPV [95% CI] |
| --- | --- | --- | --- | --- | --- |
| GI health | Concern | 57 | 57 | 0 | 1.000 [0.937, 1.000] |
| GI health | Benefit | 5 | 4 | 1 | 0.800 [0.376, 0.964] |
| Skin and coat | Concern | 58 | 57 | 1 | 0.983 [0.909, 0.997] |
| Skin and coat | Benefit | 22 | 20 | 2 | 0.909 [0.722, 0.975] |
| Ear and eye | Concern | 26 | 26 | 0 | 1.000 [0.871, 1.000] |
| Ear and eye | Benefit | 15 | 15 | 0 | 1.000 [0.796, 1.000] |
| Allergy and intolerance | Concern | 58 | 58 | 0 | 1.000 [0.938, 1.000] |
| Allergy and intolerance | Benefit | 7 | 6 | 1 | 0.857 [0.487, 0.974] |
| Weight and body condition | Concern | 58 | 57 | 1 | 0.983 [0.909, 0.997] |
| Weight and body condition | Benefit | 24 | 11 | 13^a^ | 0.458 [0.279, 0.649] |
| Energy and behavior | Concern | 52 | 43 | 9 | 0.827 [0.703, 0.906] |
| Energy and behavior | Benefit | 25 | 13 | 12^a^ | 0.520 [0.335, 0.700] |
| Dental and oral | Concern | 19 | 18 | 1 | 0.947 [0.754, 0.991] |
| Dental and oral | Benefit | 14 | 12 | 2 | 0.857 [0.601, 0.960] |
| Appetite and fussy | Concern | 46 | 46 | 0 | 1.000 [0.923, 1.000] |
| Appetite and fussy | Benefit | 8 | 6 | 2 | 0.750 [0.409, 0.929] |
| General health and illness | Concern | 50 | 48 | 2 | 0.960 [0.865, 0.989] |
| General health and illness | Benefit | 3 | 2 | 1 | 0.667 [0.208, 0.939] |
| Micro-average | All | 547 | 499 | 48 | 0.912 [0.886, 0.933] |
| Micro-average | Concern | 424 | 410 | 14 | 0.967 [0.945, 0.980] |
| Micro-average | Benefit | 123 | 89 | 34 | 0.724 [0.639, 0.795] |

^a^Higher false positive rates in weight and body condition benefits, and energy and behavior benefits were due to the overlap in how benefit-related terms were used in different contexts in owner social media posts. These categories were retained as category classification was correct, although benefit polarity was less consistent due to variability in owner language and as an exploratory analysis, results were interpreted with caution.

Table S6. Owner-reported health-related sign mentions by category and polarity in social media posts from the kibble/canned diets owner posts dataset (n = 8040), classified using an exploratory text-based health effect dictionary classifier. Values represent the number of posts (n) and percentage of total posts in which each health domain was identified with the specified polarity. “Concern only” indicates posts containing at least one concern-coded term and no benefit-coded terms; “Benefit only” indicates posts containing at least one benefit-coded term and no concern-coded terms; “Both” indicates posts containing both concern- and benefit-coded terms within the same domain; “Any” represents all posts with any health effect mention in the domain (sum of concern only, benefit only and both).

| Health effect | Concern only n (%) | Benefit only n (%) | Both n (%) | Any n (%) |
| --- | --- | --- | --- | --- |
| Gastrointestinal health | 1961 (24.4) | 196 (2.4) | 129 (1.6) | 2286 (28.4) |
| Allergy/Intolerance | 1699 (21.1) | 104 (1.3) | 129 (1.6) | 1932 (24.0) |
| Appetite/Fussy eating | 1048 (13.0) | 42 (0.5) | 21 (0.3) | 1111 (13.8) |
| Skin/Coat | 669 (8.3) | 174 (2.2) | 75 (0.9) | 918 (11.4) |
| General health/Illness | 722 (9.0) | 34 (0.4) | 7 (0.1) | 763 (9.5) |
| Weight/Body condition | 485 (6.0) | 165 (2.1) | 68 (0.8) | 718 (8.9) |
| Energy/Behavior | 145 (1.8) | 266 (3.3) | 17 (0.2) | 428 (5.3) |
| Ear/Eye | 133 (1.7) | 15 (0.2) | 8 (0.1) | 156 (1.9) |
| Dental/Oral | 36 (0.4) | 18 (0.2) | 2 (0.0) | 56 (0.7) |

Table S7. Owner-reported health-related sign mentions by category and polarity in social media posts from the FD owner posts dataset (n = 924). Posts were classified using an exploratory text-based health effect dictionary classifier. Values represent the number of posts (n) and percentage of total posts in which each health domain was identified with the specified polarity. “Concern only” indicates posts containing at least one concern-coded term and no benefit-coded terms; “Benefit only” indicates posts containing at least one benefit-coded term and no concern-coded terms; “Both” indicates posts containing both concern- and benefit-coded terms within the same domain; “Any” represents all posts with any health effect mention in the domain (sum of concern only, benefit only and both). FD, fresh diets including human-grade, gently/mildly cooked or home-cooked diets.

| Health effect | Concern only n (%) | Benefit only n (%) | Both n (%) | Any n (%) |
| --- | --- | --- | --- | --- |
| Gastrointestinal health | 237 (25.6) | 23 (2.5) | 22 (2.4) | 282 (30.5) |
| Allergy/Intolerance | 168 (18.2) | 8 (0.9) | 18 (1.9) | 194 (21.0) |
| Appetite/Fussy eating | 125 (13.5) | 7 (0.8) | 1 (0.1) | 133 (14.4) |
| Skin/Coat | 78 (8.4) | 26 (2.8) | 10 (1.1) | 114 (12.3) |
| General health/Illness | 120 (13.0) | 4 (0.4) | 1 (0.1) | 125 (13.5) |
| Weight/Body condition | 59 (6.4) | 22 (2.4) | 9 (1.0) | 90 (9.7) |
| Energy/Behavior | 20 (2.2) | 35 (3.8) | 2 (0.2) | 57 (6.2) |
| Ear/Eye | 20 (2.2) | 1 (0.1) | 1 (0.1) | 22 (2.4) |
| Dental/Oral | 12 (1.3) | 2 (0.2) | 3 (0.3) | 17 (1.8) |
